# Selection of key indicators for European policy monitoring and surveillance for dietary behaviour, physical activity and sedentary behaviour

**DOI:** 10.1186/s12966-021-01111-0

**Published:** 2021-04-01

**Authors:** Lina Garnica Rosas, Gert B. M. Mensink, Jonas D. Finger, Anja Schienkiewitz, Stefanie Do, Maike Wolters, Isobel Stanley, Karim Abu Omar, Katarzyna Wieczorowska-Tobis, Catherine B. Woods, Celine Murrin, Wolfgang Ahrens, Antje Hebestreit, on behalf of the PEN Consortium

**Affiliations:** 1 Robert Koch Institute, General-Pape Straße 62-66, 12101 Berlin, Germany; 2 Leibniz Institute for Prevention Research and Epidemiology – BIPS, Bremen, Germany; 3 School of Public Health, Physiotherapy and Sports Science, University College Dublin, Dublin, Ireland; 4 Friedrich-Alexander University, Erlangen, Germany; 5 Poznan University of Medical Sciences, Poznan, Poland; 6 University of Limerick, Limerick, Ireland; 7 University of Bremen, Bremen, Germany

**Keywords:** Monitoring, Surveillance, Health indicators, Obesity prevention, Policy evaluation

## Abstract

**Background:**

A pan-European approach to evaluate policy impact on health behaviour requires the employment of a consensus set of established and relevant indicators.

**Methods:**

As part of the Joint Programming Initiative on a Healthy Diet for a Healthy Life, the Policy Evaluation Network PEN identified key indicators of health behaviours and their determinants. These key indicators are already, or have the potential to be, adopted by large European Union surveillance systems for the assessment of policy impact. The iterative selection process included consultations in two rounds via email prior to a 2-days expert workshop. The experts collated a list of dietary behaviour, physical activity and sedentary behaviour indicators for European policy monitoring in young and adult populations based on existing frameworks and literature reviews. The expert panel was composed of researchers, policy makers and representatives of major European surveillance systems and related initiatives, as well as, representatives of organisations providing monitoring data, such as the European Commission and Eurostat.

**Results:**

The process provided two lists of key indicators including 37 diet ‘policy’ indicators and 35 indicators for dietary behaviour and their ‘determinants’; as well as 32 physical activity ‘policy’ indicators and 35 indicators for physical activity, sedentary behaviour and their ‘determinants’.

**Conclusion:**

A total of 139 key indicators related to the individual, the setting and the population level, and suitable for the assessment of dietary behaviour, physical activity and sedentary behaviour were prioritised by policy makers and researchers with the ultimate aim to embed policy evaluation measures in existing surveillance systems across the European Union. In a next step, data sources and suitable instruments will be identified to assess these key indicators.

**Supplementary Information:**

The online version contains supplementary material available at 10.1186/s12966-021-01111-0.

## Background

Globally, non-communicable diseases (NCDs) —mainly cardiovascular diseases, cancers, chronic respiratory diseases, and diabetes— contribute substantially to the global burden of disease [1]. NCDs are responsible for more than 40 million deaths every year, of which nearly 40% occur at a premature age, between 30 and 69 years [2]. Furthermore, they place a large burden on national health, societal and economic systems [3].

A balanced diet, a sufficient level of physical activity, and less sedentary behaviour across the life course are the most relevant factors for the prevention of NCDs and premature deaths [4]. The composition of a balanced diet can vary widely and will additionally depend on individual characteristics (e.g. age, gender, and lifestyle), cultural context, locally available foods and dietary customs. Physical activity involves any bodily movement that is produced by the contraction of the skeletal muscles and that substantially increases energy expenditure [5]. The World Health Organisation (WHO) provides basic recommendations for diet and physical activity, which have demonstrated positive health outcomes [6, 7]. Sedentary behaviour has been defined as any waking behaviour characterised by an energy expenditure ≤1.5 metabolic equivalents, such as sitting, reclining or lying down. Sedentary behaviour usually encompasses screen time (such as watching television, playing video games, e-reading, use of computer), driving a car, and reading [8].

The majority of populations are not following recommended modifications of health behaviours to achieve health benefits [9]. This constitutes a major public health concern, which has been addressed during the last decades with the development of different interventions. Most of these interventions have targeted only individual behaviours; such downstream interventions have often shown limited effect. On the contrary, systemic approaches leading to a positive food and physical activity environment - also called upstream interventions -, have the potential to improve population health by influencing people’s decisions. These upstream approaches focus on policy and economic drivers that promote healthy food consumption or support physical activity in daily life. They focus on environmental drivers affecting, for instance, the food supply chain or the walking and cycling infrastructure [10, 11].

Considering the importance of prioritising and promoting a healthy lifestyle, the European Union established different upstream policy-based approaches [4, 12, 13]. Despite their existence, a knowledge gap remains regarding their impact, relevance and effectiveness [14]. The Policy Evaluation Network (PEN) aims to close this gap and to “evaluate the existing policies on dietary, physical activity and sedentary behaviour and how they influence existing health inequities” [11]. PEN is part of the Joint Programming Initiative “Healthy Diet for a Healthy Life” (JPI HDHL), and includes 28 research institutes from seven European countries and New Zealand aiming to harmonise public health surveillance systems across Europe to prevent chronic diseases [15].

At present, comparability among countries is restricted due to the lack of indicators that are measured in a standardised manner and with objective methods, such as accelerometers or fitness trackers, blood or serum measurements. In this regard, PEN will continue the work on a roadmap towards a harmonised pan-European surveillance system. This work was begun as part of the Determinants of Diet and Physical Activity (DEDIPAC) Knowledge Hub [16] proposing a stepwise approach towards a cross-country harmonisation of health policy indicators [17]. The harmonisation of surveillance data on key indicators at individual, setting and population level as well as the identification and sharing of existing inter-sectoral health and consumer data will thus improve the assessment of the policy impact.

While current European monitoring and surveillance systems comprise an abundance of data to monitor lifestyle behaviours, there is still insufficient information for monitoring policy approaches. A first step towards the evaluation of policies is to agree upon a set of indicators of dietary, physical activity and sedentary behaviour in young and adult populations. Prioritising key indicators is valuable to evaluate the current situation and to identify progress and setbacks. Hence, these sets of indicators include 1) Policies defined as ‘decisions, plans and actions that are enforced by national or regional governments or their agencies (including at the local level) which may directly or indirectly achieve specific health goals within a society’ [11], 2) behavioural determinants (at the environmental, interpersonal and individual level), defined as variables that have been found to influence health behaviour, such as life circumstances or inequities and 3) behavioural outcome indicators.

The current work combines the physical activity and sedentary behaviour indicators in one category. This is justified, considering that these two constructs are not independent; reducing sedentary behaviour through the promotion of occasional physical activity (for example, standing, climbing stairs, short walks) can support individuals to gradually increase their levels of physical activity, towards reaching the recommended levels [12]. In addition, the current evidence shows that the availability of sedentary behaviour measurements within the European monitoring and surveillance system is scarce compared to measurements of physical activity [18] and the simultaneous collection of physical activity and sedentary behaviour indicators provides further information.

This paper summarises the process to prioritise and agree on the set of PEN key indicators for dietary, physical activity and sedentary behaviour, and their upstream determinants. This achievement corresponds to the first step towards an indicator mapping procedure, the next step within this arm of the PEN project.

## Methods

**Fig. 1:**
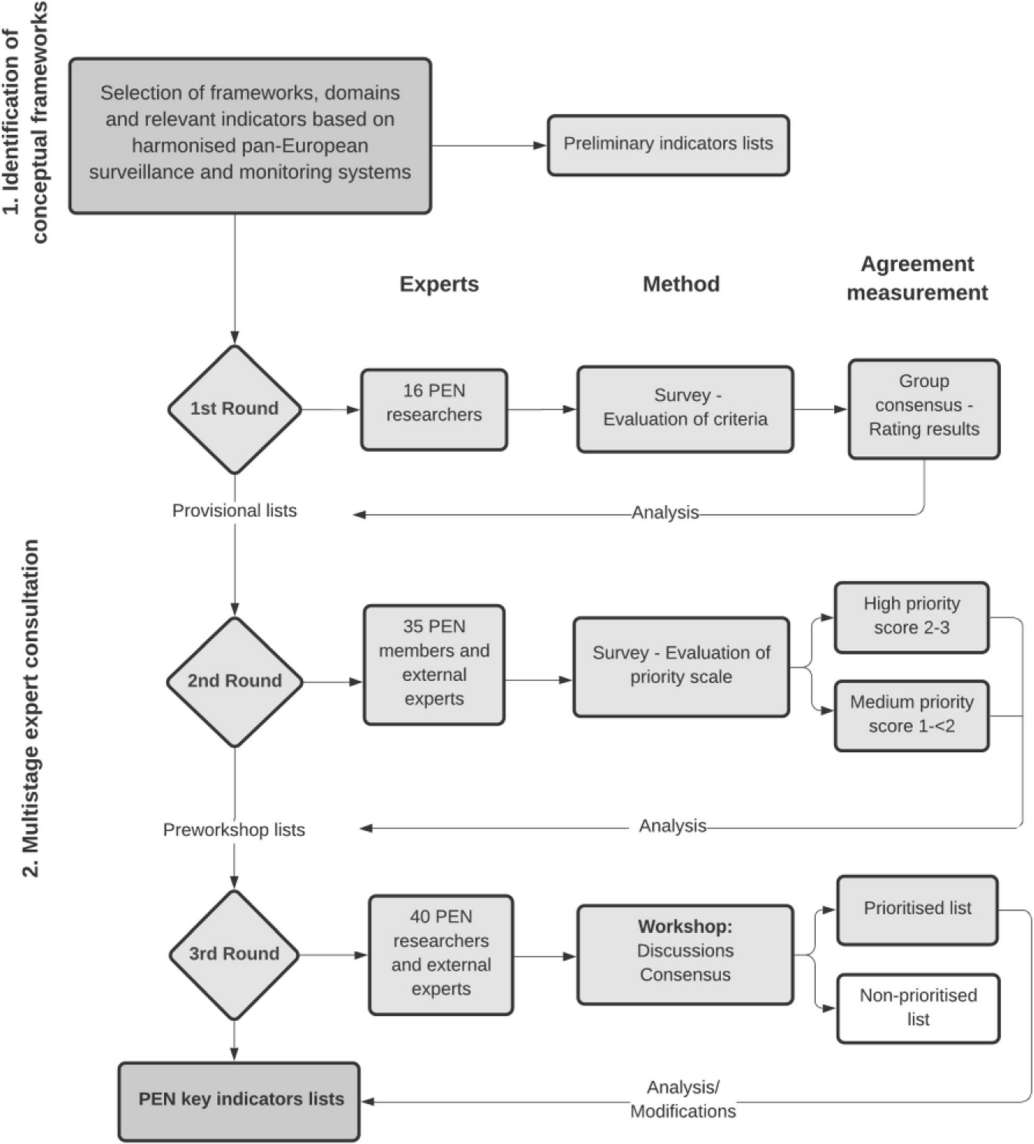
Summary of the process to select the PEN key indicator list

Overall, prioritisation of key indicators was accomplished following an iterative process. At the beginning, existing frameworks and literature reviews were identified to collate a preliminary list. During the next phase, a consultation process was organised in three rounds, involving PEN researchers and external experts. Figure 1 shows the stages of the selection process.

### Literature scan

We scanned recent literature reviews based on tacit knowledge of the research team a) to identify relevant frameworks with a focus on health promotion and obesity prevention as a starting point for collating comprehensive indicator lists as well as b) to add indicators that are associated with dietary behaviour, physical activity and/or sedentary behaviour in European populations but were missing in the frameworks. The main focus was to identify all potentially important indicators. Furthermore, we intended to identify indicators for all age groups and those which are meaningful in the European context [19–22]. Indicators were included in the list if at least one study reported a significant association with dietary, physical activity or sedentary behaviour. For this step we mainly used the consolidated evidence provided by the systematic reviews from the DEDIPAC project [18, 23]. DEDIPAC provided state-of-the-art systematic literature reviews presenting up-to date evidence regarding individual, social, and environmental determinants of physical activity, dietary and sedentary behaviours in different demographic groups [16].

For policy indicators it is unfeasible to measure the direct impact on individual behaviour with traditional epidemiological methods and potentially important indicators would be missed if these were selected based on statistical significance in epidemiological studies. Therefore, we selected established frameworks and consulted experts in the PEN network to identify current initiatives that developed inventories of indicators for policy evaluation.

### Identification of conceptual frameworks

Regarding the frameworks targeting dietary behaviours counteracting obesity in Europe, we selected frameworks based on their ability to provide upstream factors and determinants for dietary behaviour but also based on the scientific soundness used for formulation and their appropriateness. The main frameworks used for the overarching domain structure, for the dietary behaviour policy indicators, were the underlying concept of the Food-Environment Policy Index from the International Network for Food and Obesity/non-communicable diseases Research, Monitoring and Action Support (INFORMAS) [24], the NOURISHING framework [25], and the Healthy and Equitable Eating (HE2) frameworks [26]. For the dietary behaviour outcomes and their determinants, the Determinants Of Nutrition and Eating (DONE) framework [27], and the Adipositas Monitoring (AdiMon) population-wide monitoring system [28] were selected. The DONE framework was established in the DEDIPAC project and describes well recognized population-level determinants of diet. AdiMon systematically compiles and provides population-wide data on factors determining obesity, and health promotion measures. Since the purpose of this first step was to provide the invited experts with an extensive list of all potentially important indicators to be used for selection and further prioritisation steps, we included all indicators from the above listed frameworks - also those with minor evidence for a direct association with dietary behaviour. As an exception, we did not include the policy level indicators from the DONE framework, since these were covered in more detail by the more recent INFORMAS, NOURISHING and HE2 frameworks.

Four main frameworks were selected for physical activity and sedentary behaviour policy evaluation on the same criterion as explained above.

For the physical activity policy indicators, we referred to the WHO’s Global Action Plan on Physical Activity (GAPPA) [12], the Comprehensive Analysis of Policy on Physical Activity (CAPPA) framework [29], the MOVING framework [30], and the protocol of WHO Health Enhancing Physical Activity Policy Audit Tool (HEPA-PAT) [31]. GAPPA describes an overall concept of government approaches to reduce physical inactivity and sedentary behaviour. CAPPA identifies six building blocks for policy analysis. MOVING was developed within the Confronting Obesity: Co-creating policy with youth project (CO-CREATE) [32] and includes policy indicators that are closely related to the dimensions of the GAPPA framework. The HEPA-PAT provides a set of policy indicators across 11 sections. For the physical activity and sedentary behaviour outcomes and their determinants we consulted the model of physical activity correlates [33], the AdiMon population-wide monitoring system [28], the Eurostat Database [34], the Special Eurobarometer on sport and physical activity [35], the European Union Physical Activity and Sport Monitoring System (EUPASMOS) [36], the Health at a Glance – Organisation for Economic Co-operation and Development (OECD) Indicators [37], the Health Behaviour in School-aged Children surveillance system (HBSC) [38], the Information for Action (INFACT) Joint Action on Health Information [39], and the Science and Technology in childhood Obesity Policy (STOP) [40].

Based on this information we compiled a preliminary list containing the potentially important dietary, physical activity and sedentary behaviour indicators for European policy monitoring in young and adult populations considering associations between upstream factors and behaviours from scientific research. This list served as the basis for the subsequent consultation steps, which focussed on expert judgement and consensus about the importance of indicators for policy evaluation.

### Multistage expert consultation

We conducted three rating rounds aiming at: 1. the collection of experts’ opinion and advice regarding the indicator ranking; 2. the reduction of the number of indicators, through an iterative process based on expert agreement; and 3. consensus about the final key indicators. This stage of the process started with the identification of experts, who were then invited to the consultation rounds individually. The first and second rounds were performed with a Delphi-like technique [41], while the last one was completed in face-to-face discussions during a 2-day workshop. In addition, key socio-demographic, economic and equity indicators were added to the priority list to facilitate the evaluation of policy impact on vulnerable groups.

Generally, the selection and prioritisation of key indicators were based on the published conceptual framework [17] envisaging the establishment of a harmonised pan-European surveillance and monitoring system. In this regard, harmonisation was defined as the process of minimising differences in measures, variables and methods, so that measured parameters are comparable across countries [17].

#### Identification of experts

The expert panel was composed of PEN researchers and external experts, all with substantial knowledge in the fields of dietary, physical activity and sedentary behaviour at the European level. The first consultation round was completed by 16 researchers, who were involved in the PEN project, the second round by 35 experts (27 PEN researchers and 8 external experts) and the last round included a total of 40 researchers and experts (25 PEN consortium researchers and 15 external experts).

For the second and third rating/consultation rounds, external experts were invited to participate on a voluntary basis. As this process built upon an earlier study during the DEDIPAC project, we invited representatives of European surveillance and monitoring systems which were identified in a recently published inventory [18]. For these systems, experts were involved in the consultation for DEDIPAC [17] and re-invited for the present work: HBSC [38], the WHO Childhood Obesity Surveillance Initiative (COSI) [42], the European Health Interview Survey (EHIS) [34, 43], and the German Health Interview and Examination Survey for Children and Adolescents (KIGGS) [44]. Other external experts were representatives of organisations providing monitoring data (WHO, OECD, European Commission - Directorate-General Sante and Eurostat -, World Cancer Research Fund). The experts were supplemented with representatives from current European Union projects in this field such as Science and Technology in childhood Obesity Policy (STOP) [40] and CO-CREATE [32]. The experts came from 10 European countries (Belgium, Denmark, England, Germany, Ireland, Luxembourg, Norway, Russian Federation, Switzerland, and The Netherlands).

#### First consultation round: collation of preliminary indicator lists

For the first consultation round, existing criteria for indicator selection published by the Public Health Agency of Canada and the Dutch National Institute for Public Health and the Environment, the Netherlands (Rijksinstituut voor Volksgezondheid en Milieu; RIVM) were adapted for the purpose of this task and experts were instructed to apply them accordingly. The criteria are listed in Table 1 and required indicators to be: 1) relevant to evaluate policies; 2) actionable to inform and influence policies; 3) meaningful and useable for analysing policy impact; 4) accurate; 5) feasible and efficient (i.e. possible to be measured in surveillance and monitoring systems); 6) ongoing (i.e. useful to collect data regularly and comparable over time); 7) internationally comparable; and 8) applicable to all age groups.

**Table 1:** Selection criteria for the first consultation round

Indicator criteria	Description
The indicator is relevant	The indicator is clearly relevant to policy evaluation of lifestyle/NCDs prevention and/or is a plausible proxy for the underlying measure.
The indicator is actionable	The indicator provides information that can lead to action for change: inform and influence policies. It is actionable in regard to the PEN case studies.
The indicator is meaningful and useable	The information must be easy to understand, relevant for governments plans and priorities and useful for public health action (e.g. targets population groups that are likely more affected)
The indicator is accurate	*Scientific soundness:* The scientific evidence supporting a link between the performance of an indicator and lifestyle change/NCDs prevention is strong.
	*Validity:* The indicator appears reasonable as a measure of what it is intended to measure (face validity), and the components of the indicator make sense (construct validity).
	*Reliability:* The same results can be obtained if measurements are repeated under identical conditions.
The indicator is feasible/efficient	Sufficient good quality data are already available and accessible, or data collection can be put in place at relatively low costs.
The indicator is ongoing	Data can be regularly collected and compared over time.
The indicator is internationally comparable	The indicator is clearly relevant to different cultural settings and regions in Europe and not entirely national context bound. The information can be harmonised across all European Union member states.
The indicator is age- independent	The indicator is applicable to all age groups.

***Abbreviations:*** *NCDs* Non-Communicable Diseases, *PEN* Policy Evaluation Network

Note: adapted from the Public Health Agency of Canada and the Dutch National Institute for Public Health and the Environment (RIVM) [45, 46]

PEN researchers rated every pre-selected indicator according to an agreement scale from − 1 to 1 point (− 1 = disagree, 0 = neutral, 1 = agree) for each criterion. The respondents also had the opportunity to provide comments regarding framework selection, general improvements, modification of definitions, missing domains, and missing indicators. The decision on which indicators to retain and which to drop was based on the overall rating points. In proportion with the total given points, we selected the indicators rated with a score of at least 8 points. Then, it was decided to compile two sets of indicator lists for the next rating procedure: one for dietary behaviour and one for physical activity and sedentary behaviour.

#### Second consultation round: prioritisation of indicator lists

In the second consultation round, the expert panel rated every indicator according to their expert opinion on its level of priority for the monitoring of lifestyle policies. They applied the following rating point scale: (0) the indicator should be removed because it is not a priority for monitoring of lifestyle/NCDs prevention policies, (1) the indicator should not be included in the priority list, a major revision is needed, (2) the indicator should be included in the priority list but it needs a minor revision, or (3) the indicator is clearly a priority for monitoring of lifestyle/NCDs prevention policies, and it should be included in the priority list without changes.

Similar to round one, additional indicators or modifications to existing indicators were suggested. Subsequently, we assembled a ranked list of indicators separated for domains, including specific comments and suggested additional indicators. Based on the average ranking scores, indicators were divided into two categories according to the level of priority: High (score 2–3) and medium (score 1- < 2). These indicators were presented at the expert workshop and served as the starting point for the prioritisation and completion during the individual ranking sessions. The indicators with low scores (less than 1) were excluded.

#### Third consultation round: the ‘Policy Evaluation Network (PEN) Expert Workshop’

The aim of the workshop was to conclude the selection and prioritisation of the indicator lists. In this round, the experts discussed and reached consensus on the suitability and completeness of the indicators previously selected. The post processing of the workshop outcome included: a) merging or re-phrasing of comparable indicators; b) excluding of indicators lacking a clear definition from the priority list and adding them to a non-priority list; and c) developing a socio-demographic, economic and equity indicators list.

## Results

**Fig. 2:**
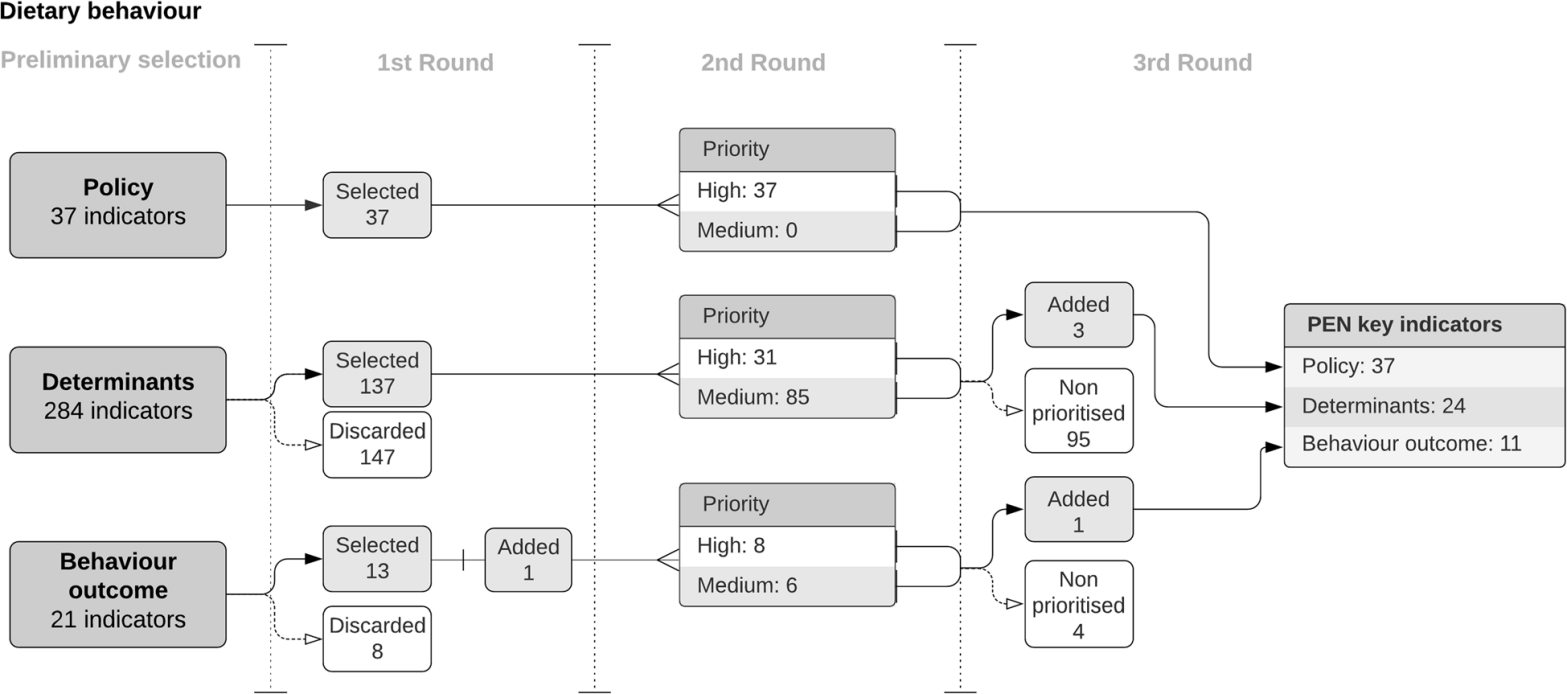
Results of the consultation rounds for dietary behaviour. Stages of the consultation rounds for dietary behaviour indicators with number of selected, added and discarded indicators

**Fig. 3:**
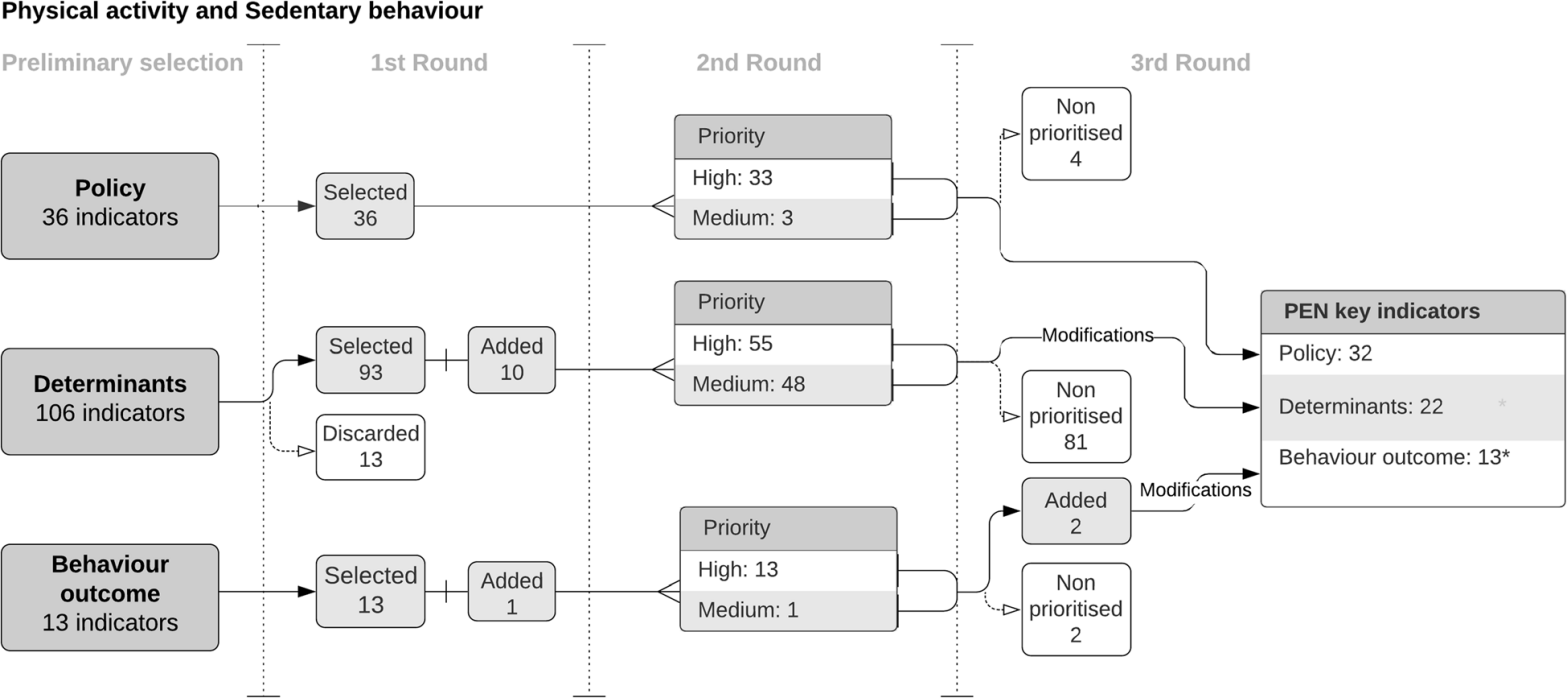
Results of the consultation rounds for physical activity and sedentary behaviour. Stages of the consultation rounds for physical activity and sedentary behaviour indicators with number of selected, added and discarded indicators. * Due to modifications performed in the physical activity and sedentary behaviour priority list including a merging of indicators, the total number of indicators was reduced

We developed an initial set of preliminary relevant indicators based on a literature review process. For dietary behaviour, we included a total of 342 indicators, consisting of 37 policy indicators, 284 determinants and 21 behaviour outcome indicators. For physical activity and sedentary behaviour, we identified 155 indicators, which included 36 policy indicators, 106 determinants and 13 behaviour outcome indicators. Figures 2 and 3 illustrate the indicator reduction process for dietary behaviour, and physical activity/sedentary behaviour, respectively.

The final PEN key indicator list for diet contained 72 indicators, including: 37 policy indicators, 24 behaviour determinant indicators and 11 behaviour outcome indicators. Regarding physical activity and sedentary behaviour, the list specifies 67 indicators including: 32 policy indicators, 22 behaviour determinant indicators (for physical environment and social environment domains), and 13 behaviour outcome indicators. In addition, a list of 17 socio-demographic, economic and equity indicators was included as determinants for stratification purposes in future analyses.

Tables 2 and 3 provide the highest ranked PEN key indicators within specific domains for dietary behaviour and physical activity/sedentary behaviour, respectively. In addition, Table 4 includes some important and recommended socio-demographic, economic and equity indicators retrieved from European monitoring systems [34, 47, 48] . The complete lists of indicators are presented in additional files 1, 2 and 3.

**Table 2:** PEN Key diet behaviour indicators with highest priority, ordered by rating score, per domain

Indicator dimension	Indicator
**Policy indicators**^**a**^ **– top ten with highest rating**	
*Prices*	Taxes or levies on healthy foods are minimised to encourage healthy food and beverage choices (e.g. low or no sales tax, excise, value-added or import duties on fruit and vegetables, subsidies).
*Composition*	Food composition targets/standards/restrictions/mandatory limits have been established and a monitoring system is in place by the government for the content of the nutrients of concern (trans fats, free sugars, salt, saturated fat, fibre) in industrially processed foods, in particular for those food groups that are major contributors to population intakes of those nutrients of concern.
*Labelling*	The government endorses one evidence informed front-of-pack labelling system containing nutritional information and interpretational aides (e.g. Nutriscore, traffic light system, keyhole) that readily allow consumers to assess a product’s unhealthiness/healthiness, and policy provisions are in place to encourage widespread uptake of endorsed system.
*Prices*	Taxes or levies on unhealthy foods and beverages (e.g. sugar-sweetened beverages, foods high in nutrients of concern) are in place and increase the retail prices of these foods to discourage unhealthy food choices where possible.
*Provision*	The government ensures that there are clear, consistent policies (including nutrition standards) which can be feasibly implemented in schools and early childhood education services for food service activities (canteens, food at events, fundraising, promotions, vending machines etc.) to provide and promote healthy food choices.
*Monitoring and evaluation*	There is regular monitoring of adult and childhood nutrition status, weight status, Body Mass Index and risk of NCDs.
*Education*	Pre-registration education curricula for all Health Care Professionals include a minimum of one nutrition module of five European Credit Transfer System or equivalent.
*Promotion*	Governmental policies are implemented to restrict commercial marketing (including sponsorship, promotion and advertisement) of unhealthy foods and beverages to children, including adolescents, in settings where children gather (e.g. preschools, schools, sports clubs and facilities and cultural events).
*Promotion*	Effective policies are implemented by the government to restrict exposure and power of promotion of unhealthy foods to children including adolescents through all media and marketing channels.
*Education*	School curricula must include knowledge and skills targets for the development of nutrition education for primary and secondary school pupils.
**Environmental determinants – top five with highest rating**	
*Extrinsic product attributes*	Relative and absolute price of healthy and unhealthy food
*Exposure to food promotion*	Exposure to food adverts for unhealthy food and beverages through all media and marketing channels.
*Portion size*	Portion size from manufacturers and food outlets in settings
*Environmental food availability and accessibility*	School food environment
*Food outlet density*	Fast food outlet density
**Interpersonal determinants (since there were only three on the prefinal list, no rating took place)**	
*Household literacy level*	Food literacy on the household level (composite score)
*Household socio-economic status*	Relative household income (household income / household size)
*Household socio-economic status*	Financial strain
**Individual determinants – top five with highest rating**	
*Anthropometrics*	Body Mass Index
*Personal socio-economic status*	Level of education
*Personal socio-economic status*	Food and nutrition insecurity
*Food beliefs*	General and relative enjoyment of healthy and unhealthy food
*Health*	Psychological/mental well-being
**Behaviour outcomes – top five with highest rating**	
*Energy, protein and fibre intake and macronutrient distribution*	Number of portions per day of pulses
*Energy, protein and fibre intake and macronutrient distribution*	Number of portions per /day of wholegrains
*Foods and beverages, groups*	Fruit intake, number of portions per day
*Foods and beverages, groups*	Vegetable intake, number of portions per day
*Foods and beverages, groups*	Sugar-sweetened beverages, glasses per day

^a^ Indicators adapted from the INFORMAS Food-Environment Policy Index [24]

**Table 3:** PEN Key physical activity and sedentary behaviour indicators with highest priority, ordered by rating score, per domain

Indicator dimension	Indicator
**Policy indicators**	
**Part 1: from MOVING and GAPPA – top five with highest rating**	
*Active environments*	Government supports the incorporation of walking and cycling infrastructure in urban, rural and transport plans.
*Active systems*	Monitoring and evaluation of policy actions - incorporating monitoring and evaluation of policy actions at the outset to ensure effect is measured.
*Active systems*	Physical activity surveillance - ensuring robust data collection on physical activity rates across the population is put in place.
*Active societies*	Government supports a national programme to promote physical activity.
*Active environments*	Government supports prioritising integrated urban design and mixed land-use policies prioritising compact, mixed-land use in urban, rural and transport plans.
**Part 2: from HEPA-PAT – top five with highest rating**	
*Collaboration HEPA (HEPA-PAT Question 4)*	Are any mechanisms or agencies in place in your country to ensure cross-sectoral collaboration on the delivery of HEPA policy, at the national level? If yes, briefly describe. Please provide information on who is involved, who is leading these efforts, and how these collaborations function in practice. Please also mention (to the extent possible) any positive or more difficult experiences. This may also include examples of collaboration with the private and voluntary sectors.
*Surveillance system for physical activity (HEPA-PAT Question 20)*	Does your country have a health surveillance or monitoring system that includes measures of physical activity or sedentary behaviour? If yes, please provide details according to age group (you may copy and paste as many response sections as needed). Please describe long-term general population surveys in: Question 20a (children and young people); Question 20b (adults) and Question 20c (older adults/seniors). Please add more boxes if needed.
*Goals for physical activity prevalence*	Does your country have any national goals (or national targets) for population prevalence of physical activity? If yes, please provide details of each target and the time frame. Please start with the most specific and measurable targets, followed by a listing or summary statement of any more general targets and goals for physical activity-related behaviours.
*National physical activity recommendations (HEPA-PAT Question 17a)*	Does your country have any national recommendations on physical activity and health? National recommendations refer to a consensus statement on how much activity is required for health benefits. If recommendations exist for any of the target groups listed, please provide details for the population subgroups (where applicable), including issuing body, year of publication, title of the document, and provide a web link if available (please also specify whether the document is available in English). If no recommendations exist, please mark the “no” column for the respective target group. If your country has officially adopted or endorsed international recommendations (e.g. of WHO or the United States Department of Health), this should be mentioned as part of the description of the respective recommendations.
*Funding of HEPA policy (HEPA-PAT Question 24a)*	Within each of the sectors listed, is funding specifically allocated or “ring-fenced” for the delivery of physical activity- related policy or action plans at the national level? Please tick yes/no, and provide the amount (and currency), if known. Please also indicate whether this funding is recurrent; that is, provided on a regular basis (e.g. annually).
**Environmental determinants – top five with highest rating**	
*Availability/ Quality/ Condition*	Availability and quality of cycling networks/paths/amenities; cycle-friendly infrastructure
*Quality/Condition/Safety*	Condition of active commuting infrastructure to and from kindergarten/school/university/work
*Availability/Proximity/ Accessibility/Quality/ Condition/Safety*	Availability and quality of parks/green space/public open space
*Availability/Quality/ Condition*	Availability and quality of footpath/sidewalks/trails
*Availability/Proximity/ Accessibility*	Availability and accessibility of public transport system
**Interpersonal determinants – top five with highest rating**	
*Supportive behaviour by friends/parents/by partner/by colleagues*	Proportion of people (all age groups) who receive significant social support from friends, colleagues, partners, parents, other relatives to be physically active
*Community support for physical activity*	Proportion of people who are aware of physical activity programmes and physical activity events organised by the community
*Supportive behaviour by educators/teachers*	Proportion of young people who receive supervision from educators/teachers to be physically active
*Support by the employer*	Proportion of people who are aware of physical activity programmes or courses offered by the employer
*Physical activity with parents*	Proportion of children who conduct physical activity with their parents at least one hour per week (AdiMon D1.12)
**Behaviour outcomes – top five with highest rating**	
*Total physical activity level*	Total time spent with physical activity per week.
*Domain-specific sedentary behaviour*	Sitting time at work/in kindergarten/school/university, during transportation in a car/bus and in leisure-time.
*Transportation-related walking/Active way to school/kindergarten*	Time spent walking in order to get to and from places in a typical week.
*Transportation-related cycling/Active way to school/kindergarten*	Time spent cycling in order to get to and from places in a typical week.
*Work-related physical activity*	Measurement of the work-related physical activity level according to different levels of physical effort.

MOVING: A policy monitoring tool for physical activity created as part of CO-CREATE project [30]. These indicators were taken from this tool

*AdiMon* A population-wide system to monitor the factors relevant to childhood obesity, created by the Robert Koch Institute [28]

***Abbreviations:*** *GAPPA* Global Action Plan on Physical Activity 2018–2030: more active people for a healthier world [12]. WHO Conceptual Framework. HEPA-PAT: Health Enhancing Physical Activity-Policy Audit Tool [31]. WHO Protocol and method for the compilation of country level policy responses

**Table 4:** Selection of suggested PEN key socio-demographic, economic, and equity indicators

Indicator dimension	Indicator
**Individual level**^**a**^ **– Recommended for stratified analyses of determinants and outcomes**	
Age	Age in completed years
Level of education	Level of education according to ISCED 2011
Sex	Sex: Male, Female, other
Employment status	Main activity status: employed, unemployed, retired, unable to work due to long standing health problems, student/pupil, fulfilling domestic tasks, compulsory military or civilian service, other [34, 47].
Migration background	People having migration background
**Societal / member states level** ^**b**^	
Risk of poverty or social exclusion rate	Sum of persons who are: at risk of poverty or severely materially deprived or living in households with very low work intensity.
Income quintile ratio	The ratio of total income received by the 20% of the population with the highest income (top quintile) to that received by the 20% of the population with the lowest income (lowest quintile). Income must be understood as equivalised disposable income*.
Gini coefficient	Defined as the relationship of cumulative shares of the population arranged according to the level of equivalised disposable income*, to the cumulative share of the equivalised total disposable income received by them.
Gross domestic product (GDP)	It is a basic measure of the overall size of a country’s economy. Equal to the sum of the gross value added of all resident institutional units engaged in production, plus any taxes on products and minus any subsidies on products. Gross value added is the difference between output and intermediate consumption.
Employment rate	The percentage of employed persons in relation to the comparable total population. For the overall employment rate, the comparison is made with the population of working-age; but employment rates can also be calculated for a particular age group and/or gender in a specific geographical area (for example the males of age 15–24 employed versus total in one European Union Member State).

^a^ Indicators retrieved from Eurostat [47] and the European Health Interview Survey (EHIS) [34, 47]

^b^ Indicators retrieved from the Portfolio of European Union social indicators for the monitoring of progress towards the objectives for social protection and social inclusion [48]

(*) The **equivalised disposable income** is the total income of a household, after tax and other deductions, that is available for spending or saving, divided by the number of household members converted into equalised adults

***Abbreviations:*** *ISCED* International Standard Classification of Education

## Discussion

### Suitability of PEN key indicators for monitoring and surveillance systems

According to the Expert Group on Health Information (EGHI) – a joint action of the European Commission and the member states – indicators suitable for health promotion should, amongst others: build on existing indicator systems; already be in use as widely as possible; focus on major public health problems and on the best potentials for effective policies, both at the European Union and at Member State levels [46]. In general, the selected PEN indicators meet these requirements.

During the process to attain the preliminary list of indicators, we considered associations with health-related behaviours or health outcomes from research studies. To reduce the preliminary list to a manageable amount, the consultation rounds considered completeness, relevance and suitability.

Thus, the final lists provide numerous indicators suitable for health surveillance and monitoring across age groups and countries in a harmonised manner. Most importantly, they provide not only indicators on health behaviours (dietary, physical activity and sedentary behaviour) and their determinants (e.g. inequality), also on behaviour outcomes (e.g. Body Mass Index), and upstream indicators (e.g. policy indicators).

### Improving the monitoring and surveillance of dietary, physical activity and sedentary behaviour

To date, monitoring and surveillance systems have focused on the evaluation of “downstream” interventions that targeted individual health behaviour (e.g. health education programmes). Nevertheless, the interventions focussing only on health behaviour, without accounting for the political, economic, physical, social, and cultural environments have shown limited impact and poor sustainability [10, 11]. Thus, current research focuses on systematic approaches (‘upstream’ interventions), demanding an evidence-based approach to improve healthy dietary behaviour and physical activity [11]. The first step into this process should include the positively or negatively impact evaluation of existing policies, affecting food policy and physical activity environments in European countries [17]. This highlights the importance of prioritising policy indicators as listed in Tables 2, 3 and 4.

To consider indicators of food policy the Food Environment Policy Index from the International Network for Food and Obesity/non-communicable diseases Research, Monitoring and Action Support (INFORMAS) was used a starting point. Policy indicators of high priority were prioritised from the domains: ‘food prices’, ‘food composition’, ‘labelling’, ‘provision’, ‘promotion’, ‘retail’, ‘trade’, ‘leadership’ and ‘governance’. The Food-Environment Policy Index - with its incorporated indicators - is a powerful tool to evaluate the level of food policies’ implementation and to benchmark against best practices in order to enhance the healthiness of food environments [24]. Food policy indicators are essential to evaluate policies, especially those that increase the ‘price of unhealthy food’ and decrease the ‘price of healthy food’. Although these policies have received attention in recent years, there is still a lack of comprehension on their effectiveness [10, 49]. Further research is needed to strengthen the evidence. Upstream indicators related to the social environment are of particular relevance for younger persons, for instance the ‘availability and accessibility of un-/healthy foods/drinks in schools’. It has been recognised that the ‘school environment’ and ‘food adverts’ influences food choices in children and adolescents and ultimately affects dietary behaviour in adulthood [50–52]. On an interpersonal level, ‘food literacy at household level’ or financial aspects were listed, however these were not rated since the number was already small and all of these indicators were included in the final list.

On individual level, a list of indicators applicable for both diet and physical activity/sedentary behaviour were prioritised. However, a few indicators were rated particularly high for diet: level of education and ‘food and nutrition insecurity’ - a proxy-measure for socio-economic or equity status [53]. Indicators of high priority for behaviour outcomes were, for instance, ‘number of portion/day’ for pulses, wholegrains, fruits and vegetables. Dietary patterns characterized by high intakes of those food groups seem to decrease the obesity risk already at young age [54]. Future health policies have to address these indicators and require the inclusion of such indicators in monitoring and surveillance systems.

To consider indicators of physical activity and sedentary behaviour policy, we selected indicators from the WHO’s GAPPA [12], the MOVING [25], and the WHO’s HEPA-PAT [31] policy frameworks based on criteria based expert ratings. These are standardised tools to evaluate policy action across different sectors for improving physical activity in the population. Some examples for policy areas covered and prioritised by these tools are the ‘incorporation of walking and cycling infrastructure’ in ‘transport plans’, or the ‘availability of a health surveillance or monitoring systems that regularly assesses information on physical activity and sedentary behaviour’. It is important to evaluate, if and how implemented policies modify environmental circumstances and if these changes, in return, have an impact on physical activity and sedentary behaviour.

Indicators of physical activity and sedentary behaviour determinants at different levels (individual, interpersonal, environmental) were selected for evaluating the impact of policies. Some examples of indicators given high priority were ‘social support’ [33], ‘participation in physical activity programmes or events’, ‘neighbourhood walkability’, ‘quality of parks/playgrounds’ and ‘provision of active commuting infrastructure’. These indicators have shown a positive impact on physical activity among both children and adults [19, 55] and may be useful to evaluate public policies [17, 56].

Indicators of domain-specific physical activity and sedentary behaviour levels were selected as the endpoint of the individual behaviour targeted by the policies. Regarding sedentary behaviour, indicators such as ‘sitting time’ and ´screen-time´ have received attention and priority. Their importance is also enhanced, considering reported associations between adverse health outcomes and ‘sitting time’ in adults [57]. The situation is less clear for children and adolescents [21], recent studies have shown weak and inconsistent associations between children’s ´screen-time´ and obesity [58]. In addition, a person’s sedentary lifestyle in early life stages may define their sedentary behaviour into adulthood [21]. At present, the European monitoring and surveillance systems have limited information on sedentary behaviour measurements especially for children, which raised the importance to prioritise and include both indicators in future assessments [18].

Socio-demographic, economic and equity indicators were categorised at the individual level, such as age, sex, level of education or employment status; and at the societal level, such as the Gini coefficient or the employment rate. Principally, the most relevant indicators were selected since they serve for stratification of policy related outcomes at the group or individual level (e.g. age, sex). These are routinely measured in monitoring systems. Some indicators indicate potential problems in coping with life circumstances for vulnerable groups (e.g. risk of poverty, income quintile ratio; see Table 4). These indicators are considered mainly to evaluate the impact of policies among children, older persons, ethnic minorities, low educated and low-income groups, and unemployed persons and to determine, for instance, reachability of certain groups and barriers for policy implementation. These indicators of vulnerability were deemed to be sufficiently important as not to undergo a further ranking and were all included in the final list.

### Instruments to assess PEN key indicators

As described earlier, the selection and prioritisation process was based on clear criteria focusing on the suitability, relevance and usefulness for policy analysis. The availability of instruments suitable for surveillance and monitoring purposes was a criterion of minor importance in this phase. Instead we aimed at providing comprehensive but still highly relevant dietary, physical activity and sedentary behaviour indicator lists. Suitable instruments to measure these relevant key indicators most accurately will be identified in subsequent PEN activities [11].

### Future implications of PEN key indicators for policy research and monitoring and surveillance systems

The identification of PEN key indicators is relevant for future evaluation of the effectiveness of policies as they may act as facilitators and barriers in the implementation of dietary-, and physical activity/sedentary behaviour-related policies. This can further guide researchers, policy makers and stakeholders in developing policy-related health promotion interventions and evaluating monitoring frameworks. It may require the adoption of particular PEN key indicators by existing or newly established health surveillance systems across Europe [11]. Once the suitable instruments to measure these indicators have been identified it will be necessary to assess their suitability for a given surveillance system. Certainly, before these indicators can be implemented on a larger scale for monitoring and surveillance, their feasibility, reliability and validity must be proven in methodological pilot studies.

Thus, the PEN key indicators might stimulate a call to action to European monitoring and surveillance systems, to “policy proof” their current indicators related to dietary, physical activity and sedentary behaviour. This comparison and evaluation might then result in the expansion and harmonisation of available data. European surveillance systems would improve substantially with the embedding of policy evaluation measures information.

Within the process of this work, it became apparent that the domain ‘sustainability’ was absent from existing policy frameworks. To approach the concept sustainability (i.e. policies supporting sustainable practices in food production) is important, since globally, and in Europe, food production is exceeding environmental limits or it is close to it. The governments have the challenge to implement an efficient and sustainable food system [59]. Thus, the future inclusion of this domain in selection and mapping procedures would be essential and requires also updating of the established frameworks in future steps following completion of the PEN project.

The PEN project focusses on the health situation and systems in the EU, although some of the applied frameworks have a global perspective (e.g. GAPPA). While several health problems may also need similar strategies in low- and middle-income countries (e.g. reducing obesity rates) other problems are specific to these countries (e.g. vitamin deficiencies, food availability and accessibility including safe drinking water) and monitoring systems may need a different focus. Therefore, several indicators in the final list may also be relevant for those countries and may be provided in future, but for prioritisation an adaption is needed.

### Strengths and limitations

The key strength of the presented work within the PEN project is the expert consultation-based approach used to develop and obtain a priority list of key indicators building on the work and experiences of previous projects. An advantage of the structured process – starting with an evidence-based preselection of relevant health indicators and successive prioritisation by expert consultations – is that the PEN indicators meet important criteria (Table 1) to serve future research and policy evaluation at its best. Furthermore, the expert panel agreed on indicators from multiple areas of concern, such as determinants at the individual and at the population level. The indicators were relevant to all age and gender groups, from different settings (at the place of work, school, university etc.) and minority groups (e.g. country of origin), reflecting the general and multidimensional approaches in current health research.

Furthermore, due to the pan-European perspective of PEN, we were able to consider different foci of public health policies operating in various European countries. This contributed to a broad range of understanding of concepts, objectives and terminology among experts. However, it also constituted a challenge to reaching consensus on the prioritisation of indicators.

Other challenges and limitations have to be addressed. To create the final key indicators list, we used the same selection process for dietary behaviour and physical activity/sedentary behaviour indicators. Nevertheless, during the process it became apparent that the indicators for policies or health interventions for dietary behaviour needed to focus on addressing and prioritising different aspects of domain levels (e.g. aspects of the food system) compared to those for physical activity/sedentary behaviour (like urban infrastructure). For dietary behaviour, effective policies may involve the whole food system (including food industry, retailers, regulations, and taxes) which can often generate a lot of resistance. For physical activity policies there seems to be reasonable agreement about how to change conditions and circumstances to improve physical activity levels, between health professionals and other groups in society. Often public policy attempts targeting upstream factors to improve dietary behaviour are counteracted by powerful vested interests. Examples of this are actions by a strong industry lobby against food policy measures such as introduction of sugar taxes or traffic lights as front of pack labelling. Therefore, to-date, promoting dietary change has relied heavily on changing individual behaviour. To update present frameworks in this field is recommended in order to address the new developments. This is less of an issue for promoting physical activity because industry can benefit from marketing sport products and from services promoting physical activity.

Furthermore, some formulations of indicators did not have a clear and consistent definition. However, it is important to emphasise that the main objective during this step of PEN was to select indicators based on their priority level, rather than on their exact formulation. Phrasing more precise definitions will be part of the next step that includes the mapping of available sources [11, 60].

Even though physical activity researchers consider that the concepts of physical activity and sedentary behaviour have independent impacts on health, as it was mentioned before, the current work combines these two concepts within one selection process. This may be a limitation as separate selection procedures might produce different results and could possibly extend the indicators for sedentary behaviour. This might be important for the future inclusion of relevant indicators for sedentary behaviour in monitoring and surveillance systems.

## Conclusions

In order to select and prioritise PEN key indicators for health surveillance and monitoring multiple steps had to be considered: 1) knowledge on the association between health-related behaviours, their determinants and health outcomes; 2) relevance for evaluating policy impact based on opinion of experts; and 3) usefulness for evaluating policy impact in different age and vulnerable groups. Through this process we reached a list of prioritised indicators which might be useful for researchers, policy makers interested in evaluate the current situation of dietary, physical activity and sedentary behaviour, in Europe to consult.

In the next step, PEN indicators for policy evaluation will be mapped against available European data in order to provide a searchable catalogue for researchers, policy makers and other interested stakeholders to facilitate the development and evaluation of their policy-related work [60]. This catalogue will facilitate the selection of suitable instruments to measure variables that describe relevant key indicators. The instruments will then serve the ultimate aim: the development of a protocol for the establishment of a harmonised pan-European surveillance of young and adult populations and a monitoring system that includes distal indicators driving the health behaviours of interest [17]. The assessment of comparable surveillance data on key indicators and their determinants at individual, setting and population level, and the identification of existing intersectoral health and consumer data will help to improve policy outcome and impact evaluation in Europe.

## Supplementary Information

**Additional file 1:** PEN key diet indicators list

**Additional file 2.** PEN Key physical activity and sedentary behaviour list

**Additional file 3.** PEN key socio-demographic, economic, and equity indicators list

## Data Availability

The data generated or analysed supporting the conclusions of this article is included within the article (and its additional files 1, 2, and 3).

## Abbreviations

AdiMon: Adipositas Monitoring
CAPPA: Comprehensive Analysis of Policy on Physical Activity
CO-CREATE: Confronting Obesity: Co-creating policy with youth
COSI: Childhood Obesity Surveillance Initiative
DEDIPAC: Determinants of Diet and Physical Activity
DONE: Determinants of Nutrition and Eating
ECHI: European Core Health Indicators
EGHI: Expert Group on Health Information
EHIS: European Health Interview Survey
EUPASMOS: The European Union Physical Activity and Sport Monitoring System
GAPPA: Global Action Plan on Physical Activity
GDP: Gross Domestic Product
HBSC: Health Behaviour in School-aged Children
HE2: Healthy and Equitable Eating
HEPA: Health-Enhancing Physical Activity
INFACT: Information for Action
INFORMAS: International Network for Food and Obesity/non-communicable diseases Research, Monitoring and Action Support
ISCED: International Standard Classification of Education
JPI HDHL: Joint Programming Initiative on a Healthy Diet for a Healthy Life
KIGGS: German Health Interview and Examination Survey for Children and Adolescents
NCDs: Non-Communicable Diseases
OECD: Organisation for Economic Co-operation and Development
PAT: Policy Audit Tool
PEN: Policy Evaluation Network
RIVM: Dutch National Institute for Public Health and the Environment (Rijksinstituut voor Volksgezondheid en Milieu)
STOP: Science and Technology in childhood Obesity Policy
WHO: World Health Organization

## References

[1] The Lancet (2018). GBD 2017: a fragile world. Lancet.

[2] World Health Organization. Noncommunicable diseases. 2018. https://www.who.int/mediacentre/factsheets/fs355/en/\. Accessed 6 Dec 2019.

[3] Swinburn B, Sacks G, Vandevijvere S, Kumanyika S, Lobstein T, Neal B, Barquera S, Friel S, Hawkes C, Kelly B, L'Abbé M, Lee A, Ma J, Macmullan J, Mohan S, Monteiro C, Rayner M, Sanders D, Snowdon W, Walker C, INFORMAS (2013). INFORMAS (international network for food and obesity/non-communicable diseases research, monitoring and action support): overview and key principles. Obes Rev.

[4] World Health Organization. Global action plan for the prevention and control of noncommunicable diseases 2013-2020. 2013. https://apps.who.int/iris/bitstream/handle/10665/94384/9789241506236_eng.pdf. Accessed 15 Nov 2019.

[5] Caspersen CJ, Powell KE, Christenson GM (1985). Physical activity, exercise, and physical fitness: definitions and distinctions for health-related research. Public Health Rep.

[6] World Health Organization. Healthy diet. 2020. https://www.who.int/news-room/fact-sheets/detail/healthy-diet. Accessed 15 Jun 2020.

[7] World Health Organization. Physical activity. 2018. https://www.who.int/news-room/fact-sheets/detail/physical-activity. Accessed 25 Jul 2020.

[8] Tremblay MS, Aubert S, Barnes JD, Saunders TJ, Carson V, Latimer-Cheung AE (2017). Sedentary behavior research network (SBRN) - terminology consensus project process and outcome. Int J Behav Nutr Phys Act.

[9] World health Organization. Controlling the global obesity epidemic 2020. https://www.who.int/nutrition/topics/obesity/en/. Accessed 15 Jun 2020.

[10] Swinburn BA, Sacks G, Hall KD, McPherson K, Finegood DT, Moodie ML, Gortmaker SL (2011). The global obesity pandemic: shaped by global drivers and local environments. Lancet..

[11] Lakerveld J, Woods C, Hebestreit A, Brenner H, Flechtner-Mors M, Harrington JM, Kamphuis CBM, Laxy M, Luszczynska A, Mazzocchi M, Murrin C, Poelman M, Steenhuis I, Roos G, Steinacker JM, Stock CC, van Lenthe F, Zeeb H, Zukowska J, Ahrens W (2020). Advancing the evidence base for public policies impacting on dietary behaviour, physical activity and sedentary behaviour in Europe: the policy evaluation network promoting a multidisciplinary approach. Food Policy.

[12] World Health Organization. Global action plan on physical activity 2018–2030: more active people for a healthier world. (GAPPA) Conceptual Framework 2018. https://www.who.int/ncds/prevention/physical-activity/global-action-plan-2018-2030/en/. Accessed 19 Nov 2019.

[13] World Health Organization. European Food and Nutrition Action Plan 2015–2020. 2014. https://www.euro.who.int/__data/assets/pdf_file/0008/253727/64wd14e_FoodNutAP_140426.pdf?ua=1. Accessed 25 Nov 2019.

[14] Allen LN, Nicholson BD, Yeung BYT, Goiana-da-Silva F (2020). Implementation of non-communicable disease policies: a geopolitical analysis of 151 countries. Lancet Glob Health.

[15] Joint Programming Initiative (JPI) PEN. PEN Policy Evaluation Network 2019. https://www.jpi-pen.eu/. Accessed 15 Jun 2020.

[16] Brug J, van der Ploeg HP, Loyen A, Ahrens W, Allais O, Andersen LF (2017). Determinants of diet and physical activity (DEDIPAC): a summary of findings. Int J Behav Nutr Phys Act.

[17] Hebestreit A, Thumann B, Wolters M, Bucksch J, Huybrechts I, Inchley J (2019). Road map towards a harmonized pan-European surveillance of obesity-related lifestyle behaviours and their determinants in children and adolescents. Int J Public Health..

[18] Bel-Serrat S, Huybrechts I, Thumann B, Hebestreit A, Abuja P, de Henauw S (2017). Inventory of surveillance systems assessing dietary, physical activity and sedentary behaviours in Europe: a DEDIPAC study. Eur J Pub Health.

[19] Buck C, Eiben G, Lauria F, Konstabel K, Page A, Ahrens W (2019). Urban Moveability and physical activity in children: longitudinal results from the IDEFICS and I Family cohort. Int J Behav Nutr Phys Act.

[20] Loyen A, van der Ploeg HP, Bauman A, Brug J, Lakerveld J (2016). European sitting championship: prevalence and correlates of self-reported sitting time in the 28 European Union member states. PLoS One.

[21] Verloigne M, Loyen A, Van Hecke L, Lakerveld J, Hendriksen I, De Bourdheaudhuij I (2016). Variation in population levels of sedentary time in European children and adolescents according to cross-European studies: a systematic literature review within DEDIPAC. Int J Behav Nutr Phys Act.

[22] Stok FM, Renner B, Clarys P, Lien N, Lakerveld J, Deliens T. Understanding eating behavior during the transition from adolescence to young adulthood: a literature review and perspective on future research directions. Nutrients. 2018;10(6). 10.3390/nu10060667. 10.3390/nu10060667 PMC6024552 29794986

[23] Osei-Kwasi HA, Nicolaou M, Powell K, Terragni L, Maes L, Stronks K (2016). Systematic mapping review of the factors influencing dietary behaviour in ethnic minority groups living in Europe: a DEDIPAC study. Int J Behav Nutr Phys Act.

[24] Swinburn B, Vandevijvere S, Kraak V, Sacks G, Snowdon W, Hawkes C, Barquera S, Friel S, Kelly B, Kumanyika S, L'Abbé M, Lee A, Lobstein T, Ma J, Macmullan J, Mohan S, Monteiro C, Neal B, Rayner M, Sanders D, Walker C, INFORMAS (2013). Monitoring and benchmarking government policies and actions to improve the healthiness of food environments: a proposed government healthy food environment policy index. Obes Rev.

[25] World Cancer Research Fund International. NOURISHING framework. 2018. https://www.wcrf.org/int/policy/policy-databases/nourishing-framework. Accessed 8 Oct 2019.

[26] Pescud M, Friel S, Lee A, Sacks G, Meertens E, Carter R, Cobcroft M, Munn E, Greenfield J (2018). Extending the paradigm: a policy framework for healthy and equitable eating (HE2). Public Health Nutr.

[27] Stok FM, Hoffmann S, Volkert D, Boeing H, Ensenauer R, Stelmach-Mardas M, Kiesswetter E, Weber A, Rohm H, Lien N, Brug J, Holdsworth M, Renner B (2017). The DONE framework: creation, evaluation, and updating of an interdisciplinary, dynamic framework 2.0 of determinants of nutrition and eating. PLoS One.

[28] AdiMon. The AdiMon Indicator System. Robert Koch Institute 2019. https://www.rki.de/EN/Content/Health_Monitoring/HealthSurveys/AdiMon/AdiMon_node.html. Accessed 16 Dec 2020.

[29] Klepac Pogrmilovic B, O'Sullivan G, Milton K, Biddle SJH, Bauman A, Bellew W (2019). The development of the comprehensive analysis of policy on physical activity (CAPPA) framework. Int J Behav Nutr Phys Act.

[30] World Cancer Research Fund International. MOVING Framework 2020. https://www.wcrf.org/int/policy/policy-databases/moving-framework. Accessed 15 Jul 2020.

[31] World Health Organization. Health-enhancing physical activity (HEPA) policy audit tool (PAT) 2015. https://www.euro.who.int/__data/assets/pdf_file/0010/286795/Health-enhancing_physical_activityHEPApolicy_audit_toolPATVersion_2.pdf. Accessed 15 Nov 2019.

[32] Norwegian Institute of Public Health. Co-Create study. 2018. https://www.fhi.no/en/studies/co-create. Accessed 23 Oct 2019.

[33] Bauman AE, Reis RS, Sallis JF, Wells JC, Loos RJ, Martin BW (2012). Correlates of physical activity: why are some people physically active and others not?. Lancet..

[34] European Union, Eurostat. European Health Interview Survey (EHIS wave 3). Methodological manual. 2018. https://ec.europa.eu/eurostat/documents/3859598/8762193/KS-02-18-240-EN-N.pdf/5fa53ed4-4367-41c4-b3f5-260ced9ff2f6. Accessed 10 Oct 2019.

[35] European Commission. Special Eurobarometer 472 - sport and physical activity 2018. https://ec.europa.eu/health//sites/health/files/nutrition_physical_activity/docs/ebs_412_en.pdf. Accessed 10 Oct 2019.

[36] EUPASMOS. The European Union Physical Activity and Sport Monitoring System (EUPASMOS) 2019. https://eupasmos.com/. Accessed 10 Nov 2019.

[37] OECD/European Union. Health at a Glance: Europe 2018: State of Health in the EU Cycle 2018. https://www.oecd-ilibrary.org/social-issues-migration-health/health-at-a-glance-europe-2018_health_glance_eur-2018-en. Accessed 10 Nov 2019.

[38] World Health Organization. Growing up unequal: gender and socioeconomic differences in young people’s health and well-being. Health Behaviour in School-aged Children (HBSC) Study: International Report from the 2013/2014 Survey 2016. https://www.euro.who.int/__data/assets/pdf_file/0003/303438/HSBC-No.7-Growing-up-unequal-Full-Report.pdf. Accessed 15 Nov 2019.

[39] INFACT Joint action on health information. INFACT Joint Action on Health Information 2018. https://www.inf-act.eu/. Accessed 25 Feb 2020.

[40] STOP. Science and Technology in Childhood Obesity Policy 2018. https://www.stopchildobesity.eu/what-is-stop/. Accessed 10 Oct 2019.

[41] Slade SC, Dionne CE, Underwood M, Buchbinder R (2014). Standardised method for reporting exercise programmes: protocol for a modified Delphi study. BMJ Open.

[42] World Health Organization. Childhood Obesity Surveillance Initiative (COSI). Protocol. 2017. https://www.euro.who.int/__data/assets/pdf_file/0018/333900/COSI-protocol-en.pdf?ua=1. Accessed 18 Oct 2019.

[43] Lange C, Finger JD, Allen J, Born S, Hoebel J, Kuhnert R, Müters S, Thelen J, Schmich P, Varga M, von der Lippe E, Wetzstein M, Ziese T (2017). Implementation of the European health interview survey (EHIS) into the German health update (GEDA). Arch Public Health.

[44] Kamtsiuris P, Lange M, Schaffrath RA (2007). Der Kinder- und Jugendgesundheitssurvey (KiGGS): Stichprobendesign, Response und Nonresponse-Analyse. Bundesgesundheitsbl Gesundheitsforsch Gesundheitsschutz.

[45] Betancourt M, Roberts K, Bennett T, Driscoll E, Jayaraman G, Pelletier L (2014). Monitoring chronic diseases in Canada: the chronic disease Indicator framework. Chronic Dis Inj Canada.

[46] National Institute for Public Health and the Environment (RIVM). ECHIM ECHI Indicator development and documentation. Joint Action for ECHIM Final Report Part II. 2012. https://www.volksgezondheidenzorg.info/sites/default/files/echim-final-report_part-ii_pdf.pdf. Accessed 05 Nov 2019.

[47] European Commission. Eurostat -Item 4.2 Standardisation of social variables -Progress report- Meeting of European Directors of Social Statistics: Luxembourg. 2017. https://circabc.europa.eu/sd/a/7039be8c-a45a-493f-bc49-987e0ba8f798/DSS-2017-Mar-4.2%20Standardisation%20of%20social%20variables%20%20progress%20report.pdf. .

[48] European Union. Social protection committee indicators sub-group. Portfolio of EU Social Indicators for the Monitoring of Progress Towards the EU Objectives for Social Protection and Social Inclusion 2015. https://ec.europa.eu/social/main.jsp?catId=738&langId=en&pubId=7855&furtherPubs=yes. Accessed 10 Oct 2019.

[49] Jensen JD, Smed S (2018). State-of-the-art for food taxes to promote public health. Proc Nutr Soc.

[50] Ventura AK, Worobey J (2013). Early influences on the development of food preferences. Curr Biol.

[51] Olafsdottir S, Eiben G, Prell H, Hense S, Lissner L, Marild S (2014). Young children's screen habits are associated with consumption of sweetened beverages independently of parental norms. Int J Public Health.

[52] De Cosmi V, Scaglioni S, Agostoni C (2017). Early taste experiences and later food choices. Nutrients..

[53] Borch A, Kjaernes U (2016). Food security and food insecurity in Europe: an analysis of the academic discourse (1975-2013). Appetite..

[54] Pala V, Lissner L, Hebestreit A, Lanfer A, Sieri S, Siani A, Huybrechts I, Kambek L, Molnar D, Tornaritis M, Moreno L, Ahrens W, Krogh V (2013). Dietary patterns and longitudinal change in body mass in European children: a follow-up study on the IDEFICS multicenter cohort. Eur J Clin Nutr.

[55] Smith M, Hosking J, Woodward A, Witten K, MacMillan A, Field A, Baas P, Mackie H (2017). Systematic literature review of built environment effects on physical activity and active transport - an update and new findings on health equity. Int J Behav Nutr Phys Act.

[56] Heath GW, Parra DC, Sarmiento OL, Andersen LB, Owen N, Goenka S, Montes F, Brownson RC, Lancet Physical Activity Series Working Group (2012). Evidence-based intervention in physical activity: lessons from around the world. Lancet..

[57] Loyen A, Van Hecke L, Verloigne M, Hendriksen I, Lakerveld J, Steene-Johannessen J (2016). Variation in population levels of physical activity in European adults according to cross-European studies: a systematic literature review within DEDIPAC. Int J Behav Nutr Phys Act.

[58] Biddle SJH, Bengoechea EG, Wiesner G (2017). Sedentary behaviour and adiposity in youth: a systematic review of reviews and analysis of causality. Int J Behav Nutr Phys Act.

[59] European Commission. Sustainable food. 2019. https://ec.europa.eu/environment/eussd/food.htm. Accessed 30 Jul 2020.

[60] Stanley I, Hebstreit A, Murrin C. Health surveillance indicators: what is available in European data sets for policy evaluation? Eur J of Public Health. 2020; 30 Supplement 5: ckaa165.328. 10.1093/eurpub/ckaa165.328.

