# Supplementary material for: Selection of key indicators for European policy monitoring and surveillance for dietary behaviour, physical activity and sedentary behaviour
Source: Int J Behav Nutr Phys Act. 2021 Apr 1;18:48. doi: 10.1186/s12966-021-01111-0 (PMC8015190; doi:10.1186/s12966-021-01111-0)
Supplement: Supplementary file 1 — Additional file 1: PEN key diet indicators list [file 12966_2021_1111_MOESM1_ESM.docx]

**Additional file 1 (supplementary material)**

**PEN Key diet indicators list**

The policy indicators are adapted from the following frameworks and reviews: Food-Environment Policy Index (Food-EPI) (1), the NOURISHING (2), and the Healthy and Equitable Eating (HE^2^) frameworks (3).

The dietary behaviour outcomes indicators and their determinants are adapted from: the Determinants Of Nutrition and Eating (DONE) framework (4), Bel-Serrat et al. 2017 (5), Monteiro et al. 2019 (6), and the AdiMon population-wide monitoring system (7).

**Table S1: PEN Key diet indicators – Policy**

| **Indicator domain** | **Indicator dimension** | **Indicator** |
| --- | --- | --- |
| *Policy* | *Prices* | Taxes or levies on healthy foods are minimised to encourage healthy food and beverage choices (e.g. low or no sales tax, excise, value-added or import duties on fruit and vegetables, subsidies). |
|  | *Composition* | Food composition targets/standards/restrictions/mandatory limits have been established and a monitoring system is in place by the government for the content of the nutrients of concern (trans fats, free sugars, salt, saturated fat, fibre) in industrially processed foods, in particular for those food groups that are major contributors to population intakes of those nutrients of concern. |
|  | *Labelling* | The government endorses one evidence informed front-of-pack labelling system containing nutritional information and interpretational aides (e.g. Nutriscore, traffic light system, keyhole) that readily allow consumers to assess a product’s un-healthiness/healthiness, and policy provisions are in place to encourage widespread uptake of endorsed system. |
|  | *Prices* | Taxes or levies on unhealthy foods and beverages (e.g. sugar-sweetened beverages, foods high in nutrients of concern) are in place and increase the retail prices of these foods to discourage unhealthy food choices where possible. |
|  | *Provision* | The government ensures that there are clear, consistent policies (including nutrition standards) which can be feasibly implemented in schools and early childhood education services for food service activities (canteens, food at events, fundraising, promotions, vending machines etc.) to provide and promote healthy food choices. |
|  | *Monitoring and Evaluation* | There is regular monitoring of adult and childhood nutrition status, weight status, Body Mass Index and risk of NCDs. |
|  | *Education* | Pre-registration education curricula for all Health Care Professionals include a minimum of one nutrition module of five European Credit Transfer System or equivalent. |
|  | *Promotion* | Governmental policies are implemented to restrict commercial marketing (including sponsorship, promotion and advertisement) of unhealthy foods and beverages to children, including adolescents, in settings where children gather (e.g. preschools, schools, sports clubs and facilities and cultural events). |
|  | *Promotion* | Effective policies are implemented by the government to restrict exposure and power of promotion of unhealthy foods to children including adolescents through all media and marketing channels. |
|  | *Education* | School curricula must include knowledge and skills targets for the development of nutrition education for primary and secondary school pupils. |
|  | *Inequality* | There are processes in place to ensure that population nutrition, health outcomes and reducing health inequalities or health impacts in vulnerable populations are considered and prioritised in the development of all government policies relating to food. |
|  | *Provision* | The government ensures that there are clear, consistent policies, which can be feasibly implemented, in other public sector settings for food service activities (canteens, food at events, fundraising, promotions, vending machines, public procurement standards etc.) to provide and promote healthy food choices. |
|  | *Monitoring and Evaluation* | Major programs and policies are regularly evaluated to assess their effectiveness and contributions to achieving the goals of the nutrition and health plans, including their impact on health inequalities. |
|  | *Monitoring and Evaluation* | Monitoring systems, implemented by the government, are in place to regularly monitor food environments at local, regional and national level (especially for food composition, for nutrients of concern, food marketing to children, nutritional quality of food in schools and other public sector settings and food procurement policies by public actors), against codes/guidelines/standards/ targets. |
|  | *Labelling* | Evidence-based regulations are in place for approving and/or reviewing claims on foods, so that consumers are protected against unsubstantiated and misleading nutrition and health claims. |
|  | *Platforms and interaction* | The governments work with a system-based approach with (local and national) organisations/partners/groups to improve the healthiness of food environments at a national level. |
|  | *Leadership* | Clear, interpretive, evidence-informed food-based dietary guidelines have been established and implemented. |
|  | *Promotion* | Governmental policies are implemented to support social marketing and fund campaigns to promote healthy and sustainable eating. |
|  | *Retail* | The government ensures existing support systems are in place to encourage the promotion and availability of healthy foods in food retail outlets by improving the food choice environment through, for example, framing in promotion policies, choice of shelf placement, type of food that is displayed close to the cashiers etc. |
|  | *Labelling* | Regulations have been enacted that make mandatory ingredient lists and nutrient declarations, in line with Codex recommendations, on the labels of all packaged foods. |
|  | *Governance* | Policies and procedures are implemented for using evidence and Health Impact Assessments in the development of food and nutrition policies. |
|  | *Nutrition in Health Care settings* | Nutrition counselling and advice is available in primary care settings. Including, promotion of healthy diets, prevention of obesity and NCDs and counselling and support for breastfeeding and infant and young child feeding. |
|  | *Funding and Resources* | Government funded research is targeted for improving food environments, reducing obesity, NCDs and their related inequalities. |
|  | *Inequality* | The government ensures that social support programs (e.g. food vouchers and food banks) offer healthy food and set nutrition standards. |
|  | *Inequality* | Systems are in place to regularly monitor household food and nutrition insecurity at a National level. |
|  | *Inequality* | Waste reduction policies for food retail and food service outlets are in place. |
|  | *Labelling* | A simple and clearly-visible system of labelling the menu boards of restaurant chains (and progressively all restaurants) is guided by the government, which allows consumers to interpret the nutrient quality and energy content of foods and meals on sale. |
|  | *Leadership* | There is a comprehensive, transparent, up-to-date implementation plan linked to national needs and priorities, to improve food environments, reduce the intake of the nutrients of concern to meet WHO and national recommended dietary intake levels, and reduce diet-related NCDs and ensure optimal breastfeeding and complementary feeding. |
|  | *Monitoring and evaluation* | Progress towards reducing health inequalities or health impacts in vulnerable populations and social and economic determinants of health are regularly monitored. |
|  | *Promotion* | The Government has enacted legislation to implement the International Code of Marketing of Breastmilk Substitutes and further protect infant feeding from commercial influence. |
|  | *Prices* | Taxes or levies of at least 20% on unhealthy foods (e.g. sugar-sweetened beverages, foods high in nutrients of concern) are in place to discourage unhealthy food choices where possible. |
|  | *Retail* | Zoning laws and policies are implemented to place limits on the density or placement of quick serve restaurants or other outlets selling mainly unhealthy foods in communities, particularly around schools, and/or access to these outlets (e.g. opening hours). |
|  | *Leadership* | Clear population intake targets have been established by the government for the nutrients of concern and / or relevant food groups to meet WHO and national recommended dietary intake levels. |
|  | *Funding and Resources* | There is a statutory health promotion agency in place that includes an objective to improve population nutrition, allocated with a specific budget line. |
|  | *Platforms and interaction* | There are processes (e.g. health impact assessments) to assess and consider health impacts during the development of other non-food policies. |
|  | *Retail* | Zoning laws and policies are implemented to encourage the availability of outlets selling fresh fruit and vegetables and/or access to these outlets (e.g. opening hours, frequency i.e. for markets). |
|  | *Governance* | Policies and procedures are implemented for ensuring transparency in the development of food and nutrition policies, including transparent guidelines on how to involve industry and mechanisms to safeguard against conflicts of interest and protect public’s interest. |
| ***Abbreviations:*** NCDs Non-Communicable Diseases; | | |

**Table S2: PEN Key diet indicators – Determinants**

| **Indicator domain** | **Indicator dimension** | **Indicator** |
| --- | --- | --- |
| **Environmental** |  |  |
| *Product* | *Extrinsic product attributes* | Relative and absolute price of healthy and unhealthy food |
| *Meso/Macro* | *Exposure to food promotion* | Exposure to food adverts for unhealthy food and beverages through all media and marketing channels |
| *Micro* | *Portion size* | Portion size from manufacturers and food outlets in settings |
| *Meso/Macro* | *Environmental food availability and accessibility* | School food environment |
| *Meso/Macro* | *Food outlet density* | Fast food outlet density |
| *Product* | *Intrinsic product attributes* | Nutritional composition |
| *Product* | *Extrinsic product attributes* | Nutritional information |
| *Meso/Macro* | *Environmental food availability and accessibility* | Food store access |
| *Product* | *Extrinsic product attributes* | Product package size |
| *Meso/Macro* | *Market prices* | Cost of healthier market basket |
| *Meso/Macro* | *Environmental food availability and accessibility* | Neighbourhood healthy food availability |
| **Interpersonal** |  |  |
| *Social* | *Household literacy level* | Food literacy on the household level (composite score) |
| *Social* | *Household socio-economic status* | Relative household income  (household income / household size) |
| *Social* | *Household socio-economic status* | Financial strain |
| **Individual** |  |  |
| *Biological* | *Anthropometrics* | Body Mass Index |
| *Demographic* | *Personal socio-economic status* | Level of education |
| *Demographic* | *Personal socio-economic Status* | Food and nutrition insecurity |
| *Psychological* | *Food beliefs* | General and relative enjoyment of healthy and unhealthy food |
| *Psychological* | *Health* | Psychological/mental well-being |
| *Biological* | *Physical Health* | Chronic diseases |
| *Biological* | *Situational and time constraints* | Perceived stress |
| *Biological* | *Anthropometrics* | Central obesity |
| *Biological* | *Physical Health* | Frailty |
| *Situational* | *Related Health Behaviours* | Smoking |

**Table S3: PEN Key diet indicators – Behaviour outcome**

| **Indicator domain** | **Indicator dimension** | **Indicator** |
| --- | --- | --- |
| *Behaviour* | *Energy, protein and fibre intake and macronutrient distribution* | Number of portions /day of pulses |
|  | *Energy, protein and fibre intake and macronutrient distribution* | Number of portions /day of wholegrains |
|  | *Foods and beverages, groups* | Fruit intake, number of portions per day |
|  | *Foods and beverages, groups* | Vegetable intake, number of portions per day |
|  | *Foods and beverages, groups* | Sugar-sweetened beverages, glasses per day |
|  | *Foods and beverages, groups* | Ultra-processed foods, eating frequency /day |
|  | *Early life factors* | Breastfeeding |
|  | *Dietary behaviour* | Consumption frequency /day of ultra-processed snack food |
|  | *Foods and beverages, groups* | Alcohol, number of drinks per week |
|  | *Dietary behaviour* | Meal location |
|  | *Minority group specific indicators* | Changes in eating habit |

**Supplementary material references**

1. Swinburn B, Vandevijvere S, Kraak V, Sacks G, Snowdon W, Hawkes C, et al. Monitoring and benchmarking government policies and actions to improve the healthiness of food environments: a proposed Government Healthy Food Environment Policy Index. Obesity Reviews. 2013;14:24-37.

2. World Cancer Research Fund. NOURISHING framework. <https://www.wcrf.org/int/policy/policy-databases/nourishing-framework>. Accessed 08 October 2019.

3. Pescud M, Friel S, Lee A, Sacks G, Meertens E, Carter R, et al. Extending the paradigm: a policy framework for healthy and equitable eating (HE2). Public Health Nutr. 2018;21:3477-81.

4. Stok FM, Hoffmann S, Volkert D, Boeing H, Ensenauer R, Stelmach-Mardas M, et al. The DONE framework: Creation, evaluation, and updating of an interdisciplinary, dynamic framework 2.0 of determinants of nutrition and eating. PLOS ONE. 2017;12:e0171077.

5. Bel-Serrat S, Huybrechts I, Thumann B, Hebestreit A, Abuja P, de Henauw S, et al. Inventory of surveillance systems assessing dietary, physical activity and sedentary behaviours in Europe: a DEDIPAC study. Eur J Public Health. 2017;27:747-55.

6. Monteiro CA, Cannon G, Levy RB, Moubarac JC, Louzada ML, Rauber F, et al. Ultra-processed foods: what they are and how to identify them. Public Health Nutr. 2019;22:936-41.

7. AdiMon. The AdiMon Indicator System. Robert Koch Institute.2019. <https://www.rki.de/EN/Content/Health_Monitoring/HealthSurveys/AdiMon/AdiMon_node.html>. Accessed 23 Oct 2019.
