# Supplementary material for: Selection of key indicators for European policy monitoring and surveillance for dietary behaviour, physical activity and sedentary behaviour
Source: Int J Behav Nutr Phys Act. 2021 Apr 1;18:48. doi: 10.1186/s12966-021-01111-0 (PMC8015190; doi:10.1186/s12966-021-01111-0)
Supplement: Supplementary file 2 — Additional file 2. PEN Key physical activity and sedentary behaviour list [file 12966_2021_1111_MOESM2_ESM.docx]

**Additional file 2 (supplementary material)**

**PEN Key physical activity and sedentary behaviour indicators list**

The policy indicators are mostly adapted from the following frameworks and reviews: the WHO’s Global Action Plan on Physical Activity (GAPPA) (1), the Comprehensive Analysis of Policy on Physical Activity (CAPPA) framework (2),the MOVING framework (3), and the protocol of WHO Health Enhancing Physical Activity (HEPA)-Policy Audit Tool (PAT) (4).

The physical activity and sedentary behaviour outcomes indicators and their determinants are adapted from: the model of physical activity correlates (5), the AdiMon population-wide monitoring system (6), the Eurostat Database (7), the Special Eurobarometer on sport and physical activity (8), the European Union Physical Activity and Sport Monitoring System (EUPASMOS) (9), the Health at a Glance – OECD Indicators (10), the Health Behaviour in School-aged Children surveillance system (HBSC)(11), the INFACT Joint Action on Health Information (12), and the Science and Technology in childhood Obesity Policy (STOP) (13).

**Table S1: PEN Key physical activity and sedentary behaviour indicators – Policy**

| **Indicator domain** | **Indicator dimension** | **Indicator** |
| --- | --- | --- |
| **Part 1: from MOVING and GAPPA** | | |
| *Policy* | *Active environments* | Government supports the incorporation of walking and cycling infrastructure in urban, rural and transport plans. |
|  | *Active systems* | Monitoring and evaluation of policy actions – incorporating monitoring and evaluation of policy actions at the outset to ensure effect is measured. |
|  | *Active systems* | Physical activity surveillance – ensuring robust data collection on physical activity rates across the population is put in place. |
|  | *Active Societies* | Government supports a national programme to promote physical activity. |
|  | *Active environments* | Government supports prioritising integrated urban design and mixed land-use policies prioritising compact, mixed-land use in urban, rural and transport plans. |
|  | *Active people* | Government supports schools to include physical education in school curricula. |
|  | *Active environments* | Government supports the increased provision of public transport. |
|  | *Active environments* | Government supports increasing access to public open space and green spaces in urban, rural and transport plans. |
|  | *Active systems* | Financing mechanisms to fund research, surveillance and interventions. |
|  | *Active people* | Government develops and communicates physical activity guidelines |
|  | *Active environments* | Government supports increasing road safety actions for pedestrians, cyclists etc. |
|  | *Active Societies* | Government supports financial incentives for individuals to promote physical activity. |
|  | *Active environments* | Government supports design guidelines and/or regulations for buildings that encourage physical activity. |
|  | *Active people* | Government supports the inclusion of physical activity counselling, assessment and physical activity prescriptions in health care and outpatient settings. |
|  | *Active Societies* | Government supports the inclusion of the importance of physical activity for health and the role of the health care industry in the pre- and in-service training for health professionals. |
|  | *Active environments* | Government supports the facilitation of open/green space that encourages physical activity. |
|  | *Active systems* | Interdisciplinary research funding – increased research capacity across all sectors on the rates of physical inactivity or activity and policy interventions etc. |
|  | *Collaboration HEPA (HEPA PAT Question 4)* | Are any mechanisms or agencies in place in your country to ensure cross-sectoral collaboration on the delivery of HEPA policy, at the national level? If yes, briefly describe. Please provide information on who is involved, who is leading these efforts, and how these collaborations function in practice. Please also mention (to the extent possible) any positive or more difficult experiences. This may also include examples of collaboration with the private and voluntary sectors. |
|  | *Surveillance system for physical activity (HEPA PAT Question 20)* | Does your country have health surveillance or monitoring system that includes measures of physical activity or sedentary behaviour? If yes, please provide details according to age group (you may copy and paste as many response sections as needed). Please describe long-term general population surveys in: Question 20a (children and young people); Question 20b (adults) and Question 20c (older adults/seniors). Please add more boxes if needed. |
|  | *Goals for physical activity prevalence* | Does your country have any national goals (or national targets) for population prevalence of physical activity? If yes, please provide details of each target and the time frame. Please specify in which policy document(s) listed in Question 7 these goals are stated. Please start with the most specific and measurable targets, followed by a listing or summary statement of any more general targets and goals for physical activity-related behaviours. |
|  | *National physical activity recommen-dations (HEPA PAT Question 17a)* | Does your country have any national recommendations on physical activity and health? National recommendations refer to a consensus statement on how much activity is required for health benefits. If recommendations exist for any of the target groups listed, please provide details for the population subgroups (where applicable), including issuing body, year of publication, title of the document, and provide a web link if available (please also specify whether the document is available in English). If no recommendations exist, please mark the “no” column for the respective target group. If your country has officially adopted or endorsed international recommendations (e.g. of WHO or the United States Department of Health), this should be mentioned as part of the description of the respective recommendations. |
|  | *Funding of HEPA policy (HEPA PAT Question 24a)* | Within each of the sectors listed, is funding specifically allocated or “ring-fenced” for the delivery of physical activity- related policy or action plans at the national level? Please tick yes/no, and provide the amount (and currency), if known. Please also indicate whether this funding is recurrent; that is, provided on a regular basis (e.g. annually). |
|  |  | Considering all the key physical activity policy documents listed in Question 7, please indicate which settings are included for the delivery of specific HEPA actions. Please only tick those settings in which dedicated programmes or interventions are foreseen or already under way. |
|  | *Leadership (HEPA PAT Question 2)* | Please state any agency(ies) providing leadership for HEPA promotion at the national level in your country. |
|  | *National physical activity recommenda-tions (HEPA PAT Question 17b)* | Does your country have any national recommendations on reducing sedentary behaviour? If recommendations exist for any of the target groups listed, please provide details for each of the population subgroups (where applicable), including the issuing body, year of publication, title of the document, and provide a web link if available (please also specify whether the document is available in English). If no recommendations exist, please mark the “no” column for the respective target group. |
|  | *National physical activity recommenda-tions (HEPA PAT Question 17b)* | Does your country have any national recommendations on reducing sedentary behaviour? If recommendations exist for any of the target groups listed, please provide details for each of the population subgroups (where applicable), including the issuing body, year of publication, title of the document, and provide a web link if available (please also specify whether the document is available in English). If no recommendations exist, please mark the “no” column for the respective target group. |
|  | *Implementation of policy documents (HEPA PAT Question 12)* | Do any national documents or guidelines exist that support implementation of HEPA activities at the subnational level? For example, does national policy determine what is delivered at the subnational level and, if so, is this national guidance strongly adhered to? Such guidance could include programmes, structures or funding. Or is subnational policy and activity developed and implemented largely independently from the national government? Please note: please be brief here (about 300–500 words) and include cross-references to other questions (e.g. Question 7) where relevant, to avoid repetition. |
|  | *Evaluation of HEPA policy (HEPA PAT Question 22a)* | Has your country undertaken evaluation of any of the national policies or action plans listed in Question 7? If yes, please state the title of the report, publisher and year published. Where available, please also provide a web link and indicate whether an English version/summary is available. Please provide brief details of the evaluation undertaken, what has been evaluated, the data collection methods, a summary of the results and how these were used (or not) to define new policy. |
|  | *Contents of policy documents (HEPA PAT Question 7)* | Please provide details (title, timeframe, issuing body) of the current key policy documents, legislation, strategies or action plans in your country, which outline government (and, where applicable, NGO) intention to increase national levels of physical activity (see Glossary for definitions of these terms). Please list the documents according to sector and, where available, provide a web link, indicating whether an English version or summary is available. Please provide a brief description of the general content of each policy (about 100–250 words). Please mark in the right-hand column which are the most important documents for the HEPA agenda in your country and briefly explain why these documents are deemed important. Please add/remove rows as needed. |
|  | *Target groups of HEPA policy (HEPA PAT Question 14)* | Considering all the key physical activity policy documents listed in Question 7, please indicate which population groups are targeted by specific HEPA actions. Please only tick those groups for which dedicated programmes or interventions are foreseen or already under way. |
|  | *Funding of HEPA policy (HEPA PAT Question 24b)* | Within each of the sectors listed, is funding specifically allocated or “ring-fenced” for the delivery of physical activity- related policy or action plans at the subnational level? Please tick yes/no, and provide the amount (and currency), if known. Please also indicate whether this funding is recurrent; that is, provided on a regular basis (e.g. annually). |
|  | *Policy Documents (HEPA PAT Question 6)* | Please describe any key past policy documents and past events that have led to the current context of HEPA promotion in your country. This might include legislation or recent policy documents that are now technically out of date (e.g. a previous national HEPA policy that may or may not have been extended), previous landmark legislation, or other documents such as scientific reports. Key events might include political changes, position statements or scientific events that have shaped the HEPA agenda. Please list the documents/events, provide a web link (where available), and indicate if an English version or summary is available in each case. Please add/remove rows as needed. |
|  | *Communication strategy for HEPA policy (HEPA PAT Question 15)* | Does your country have a current national communication strategy (using mass media) aimed at raising awareness and promoting physical activity? If yes, please provide details of the communication activities (e.g. posters, website, television or radio advertising, etc.) and whether these activities have a common branding or slogan (e.g. “Agita Sao Paulo” or “Find 30”). If no, has your country conducted any national communication activities in the past?. |
| MOVING: A policy monitoring tool for physical activity created as part of CO-CREATE project (3).  ***Abbreviations:***  GAPPA: Global Action Plan on Physical Activity 2018–2030: more active people for a healthier world (1). WHO Conceptual Framework.  HEPA-PAT: Health Enhancing Physical Activity-Policy Audit Tool (4). WHO Protocol and method for the compilation of country level policy responses.  NGO: Non Governmental Organization | | |

**Table S2. PEN Key physical activity and sedentary behaviour indicators – Determinant indicators**

| **Indicator domain** | **Indicator dimension** | **Indicator** |
| --- | --- | --- |
| **Physical environment** | | |
| *Home, neighbourhood, community setting* | *Availability/Quality/ Condition* | Availability and quality of cycling networks/paths/amenities; cycle-friendly infrastructure |
| *Kindergarten, school, university setting/Worksite, workplace setting* | *Quality/Condition/Safety* | Condition of active commuting infrastructure to and from kindergarten/school/university/ work |
| *Home, neighbourhood, community setting* | *Availability/Proximity/ Accessibility/Quality/ Condition/Safety* | Availability and quality of parks/green space/public open space |
| *Home, neighbourhood, community setting* | *Availability/Quality/Condition* | Availability and quality of footpath/sidewalks/trails |
| *Home, neighbourhood, community setting* | *Availability/Proximity/Accessibility* | Availability and accessibility of public transport system |
| *Home, neighbourhood, community setting* | *Availability/Accessibility* | Walk-friendly infrastructure/Walkability |
| *Home, neighbourhood, community setting* | *Availability/Proximity* | Destinations and services (shops/commercial) |
| *Home, neighbourhood, community setting* | *Number of cyclists/pedestrians injured in road traffic accidents per 100 000* | Traffic safety |
| *Home, neighbourhood, community setting* | *Availability/Proximity* | Availability of sports and exercise facilities |
| *Worksite, workplace setting/ Kindergarten, school, university setting* | *Physical activity friendly design of the building and outdoor facilities / availability: Proportion of kindergartens/schools/universities having their own outdoor activity space; Proportion of kindergartens which have an outdoor activity space of at least 100 square meters (AdiMon indicator D.1.3)* | Availability of outdoor activity space in kindergarten/school/university/workplace |
| *Worksite, workplace setting/ Kindergarten, school, university setting* | *Physical activity friendly design of the building and outdoor facilities /Availability: Proportion of kindergartens/schools/universities having their own indoor activity space; Proportion of kindergartens which have an indoor activity space of at least 100 square meters (AdiMon indicator D.1.3)* | Availability of indoor activity space in kindergarten/school/university/workplace |
| *Home, neighbourhood, community setting* |  | Street lighting |
| *Home, neighbourhood, community setting* |  | Air/Noise pollution |

| **Social environment** | | |
| --- | --- | --- |
| *Home, neighbourhood, community setting/Worksite, workplace setting* | *Supportive behaviour by friends/by parents/by partner/by colleagues* | Proportion of people (all age groups) who receive significant social support from friends, colleagues, partners, parents, other relatives to be physically active |
| *Home, neighbourhood, community setting* | *Community support for physical activity* | Proportion of people who are aware of physical activity programmes and physical activity events organised by the community |
| *Home, neighbourhood, community setting* | *Community support for physical activity* | Proportion of people who are aware of physical activity programmes and physical activity events organised by the community |
| *Kindergarten, school, university setting* | *Supportive behaviour by educators/teachers* | Proportion of young people who receive supervision from educators/teachers to be physically active |
| *Worksite, workplace setting* | *Support by the employer* | Proportion of people who are aware of physical activity programs or courses offered by the employer |
| *Home, neighbourhood, community setting* | *Physical activity with parents* | Proportion of children who conduct physical activity with their parents at least one hour per week (AdiMon D1.12) |
| *Kindergarten, school, university setting* | *Support by the educational institution* | Proportion of educational institutions offering extracurricular physical activity programs or courses |
| *Home, neighbourhood, community setting* | *Seeing others active* | Proportion of people who see others being active in their neighbourhood |
| *Worksite, workplace setting* | *Participation in physical activity programs or courses at work* | Participation in physical activity programs or courses offered in the workplace or in the respective setting (i.e. elderly homes) |
| AdiMon: A population-wide system to monitor the factors relevant to childhood obesity, created by the Robert Koch Institute (6). | | |

**Table S3. PEN Key physical activity and sedentary behaviour indicators –Behaviour outcome indicators**

| **Indicator domain** | **Indicator dimension** | **Indicator** |
| --- | --- | --- |
| *Behaviour* | *Total physical activity level* | Total time spent with physical activity per week. |
|  | *Domain-specific sedentary behaviour* | Sitting time at work/in kindergarten/school/university, during transportation in a car/bus and in leisure-time. |
|  | *Transportation-related walking/Active way to school/kindergarten* | Time spent walking in order to get  to and from places in a typical week. |
|  | *Transportation-related cycling/Active way to school/kindergarten* | Time spent cycling in order to get to and from places in a typical week. |
|  | *Work-related physical activity* | Measurement of the work-related physical activity level according to different levels of physical effort. |
|  | *Sedentary behaviour* | Screen time |
|  | *Leisure-time aerobic physical activity* | Time spend with aerobic physical activity in a typical week. |
|  | *Organised sports/Exercise participation* | Regular participation in organised sports and exercise, e.g. active member in a sports club. |
|  | *Curricular physical education in school* | Average curricular physical education time per week. |
|  | *Leisure-time muscle-strengthening physical activity* | Number of days in a typical week performing muscle-strengthening activities. |
|  | *Non-organised sports/exercise participation* | Non-organised sports/exercise participation. |
|  | *Extracurricular physical activity in school* | Average extracurricular physical activity time per week. |
|  | *Physical activity in kindergarten* | Average active play time per day in kindergarten. |

**Supplementary material references**

1. World Health Organization. Global action plan on physical activity 2018–2030: more active people for a healthier world. (GAPPA) Conceptual Framework 2018. <https://www.who.int/ncds/prevention/physical-activity/global-action-plan-2018-2030/en/>. Accessed 19 Nov 2019.

2. Klepac Pogrmilovic B, O'Sullivan G, Milton K, Biddle SJH, Bauman A, Bellew W, et al. The development of the Comprehensive Analysis of Policy on Physical Activity (CAPPA) framework. Int J Behav Nutr Phys Act. 2019;16:60.

3. World Cancer Research Fund International. MOVING Framework 2020. <https://www.wcrf.org/int/policy/policy-databases/moving-framework>. Accessed 15 Jul 2020.

4. World Health Organization. Health-enhancing physical activity (HEPA) policy audit tool (PAT) 2015. <http://www.euro.who.int/__data/assets/pdf_file/0010/286795/Health-enhancing_physical_activityHEPApolicy_audit_toolPATVersion_2.pdf>. Accessed 15 Nov 2019.

5. Bauman AE, Reis RS, Sallis JF, Wells JC, Loos RJ, Martin BW. Correlates of physical activity: why are some people physically active and others not? Lancet. 2012;380:258-71.

6. AdiMon. The AdiMon Indicator System. Robert Koch Institute.2019. <https://www.rki.de/EN/Content/Health_Monitoring/HealthSurveys/AdiMon/AdiMon_node.html>. Accessed 23 Oct 2019.

7. European Union. Eurostat. European Health Interview Survey (EHIS wave 3). Methodological manual. 2018. <https://ec.europa.eu/eurostat/documents/3859598/8762193/KS-02-18-240-EN-N.pdf/5fa53ed4-4367-41c4-b3f5-260ced9ff2f6>. Accessed 10 Oct 2019.

8. European Commission. Special Eurobarometer 472 - Sport and physical activity 2018. <https://ec.europa.eu/health//sites/health/files/nutrition_physical_activity/docs/ebs_412_en.pdf>. Accessed 10 Oct 2019.

9. EUPASMOS. The European Union Physical Activity and Sport Monitoring System (EUPASMOS) 2019. <http://eupasmos.com/>. Accessed 10 Nov 2019.

10. OECD/European Union. Health at a Glance: Europe 2018: State of Health in the EU Cycle 2018. <https://www.oecd-ilibrary.org/social-issues-migration-health/health-at-a-glance-europe-2018_health_glance_eur-2018-en>. Accessed 10 Nov 2019.

11. World Health Organization. Growing up unequal: gender and socioeconomic differences in young people’s health and well-being. Health Behaviour in School-aged Children (HBSC) Study: International Report from the 2013/2014 Survey 2016. <http://www.euro.who.int/__data/assets/pdf_file/0003/303438/HSBC-No.7-Growing-up-unequal-Full-Report.pdf>. Accessed 15 Nov 2019.

12. INFACT Joint Action on Health Information. INFACT Joint Action on Health Information. 2018. <https://www.inf-act.eu/>. Accessed 25 Feb 2020.

13. STOP. Science and Technology in Childhood Obesity Policy 2018. <http://www.stopchildobesity.eu/what-is-stop/>. Accessed 10 Oct 2019.
