# Supplementary material for: Selection of key indicators for European policy monitoring and surveillance for dietary behaviour, physical activity and sedentary behaviour
Source: Int J Behav Nutr Phys Act. 2021 Apr 1;18:48. doi: 10.1186/s12966-021-01111-0 (PMC8015190; doi:10.1186/s12966-021-01111-0)
Supplement: Supplementary file 3 — Additional file 3. PEN key socio-demographic, economic, and equity indicators list [file 12966_2021_1111_MOESM3_ESM.docx]

**Additional file 3 (supplementary material)**

**PEN key socio-demographic, economic, and equity indicators list**

The socio-demographic, economic, and equity indicators at the individual level are mostly adapted from: Eurostat (1) and the European Health Interview Survey (EHIS) (1, 2). The indicators at the societal/member states level are retrieved from: the Portfolio of European Union social indicators for the monitoring of progress towards the EU objectives for social protection and social inclusion (3) and Eurostat (4).

**Table S1. PEN Key socio-demographic, economic, and equity indicators –Individual level**

| **Indicator dimension** | **Definition example** | **Suitable as breakdown dimension for outcome indicators on individual level** |
| --- | --- | --- |
| Age | Age in completed years | Yes |
| Level of education | Level of education according to ISCED 2011 | Yes |
| Sex | Sex: Male, Female, other | Yes |
| Employment status | Main activity status: Employed, Unemployed, Retired, Unable to work due to long standing health problems, Student/pupil, Fulfilling domestic tasks, Compulsory military or civilian service, Other | Yes |
| Household income | Net equivalence income according to OECD 2011. | Yes; to calculate this index, information on household size, number and age of persons living in the household and current household income are needed ^a^. |
| Household size | Number and age of persons living in a household | Yes |
| Migration background^b^ | People having migration background | Yes. According to core social variables migration background can be determined using the following variables:  -Country of birth  -Country of main citizenship  -Country of birth of the father  -Country of birth of the mother  -Country of residence  -Duration of stay in the country of residence in completed years |
| Degree of urbanisation | Degree of urbanisation | Yes |
| At risk of poverty | Defined as equivalised disposable income (after social transfer) below the - at risk-of-poverty threshold, which is set at 60 % of the national median equivalised disposable income^a^ after social transfers. | Yes |
| ^a^ The **equivalised disposable income** is the total income of a household, after tax and other deductions, that is available for spending or saving, divided by the number of household members converted into equalised adults; household members are equalised or made equivalent by weighting each according to their age, using the so-called modified [OECD](https://ec.europa.eu/eurostat/statistics-explained/index.php?title=Glossary:OECD) equivalence scale (5).  ^b^ The construction of the indicator **"migration background"** is very complex. It includes not only people who have immigrated themselves (first generation) also their children (second generation) or grandchildren (third generation). All of them might have their original citizenship or the host country citizenship or both. Furthermore, one of the parents could be an immigrant the other not. Therefore, Eurostat proposes variables (see list) to calculate an indicator on migration background.  ***Abbreviations:*** EU, European Union; ISCED, International Standard Classification of Education; OECD, Organisation for Economic Co-operation and Development | | |

**Table S2. PEN Key socio-demographic, economic, and equity indicators – Societal/member states level**

| **Indicator dimension** | **Main definition** | **Additional explanation** |
| --- | --- | --- |
| Risk of poverty or social exclusion rate | **Sum of persons who are:** at risk of poverty or severely materially deprived or living in households with very low work intensity. | Persons are counted only once even if they are affected by more than one of these phenomena:  •**Persons are considered to be at risk of poverty** after social transfers, if they have an equivalised disposable income below the risk-of-poverty threshold, which is set at 60 % of the national median equivalised disposable income. (*) •**Severely materially deprived persons** have living conditions severely constrained by a lack of resources, they experience at least 4 out of 9 following deprivations items: cannot afford i) to pay rent or utility bills, ii) keep home adequately warm, iii) face unexpected expenses, iv) eat meat, fish or a protein equivalent every second day, v) a week holiday away from home, vi) a car, vii) a washing machine, viii) a colour television, or ix) a telephone. •**People living in households with very low work intensity** are those aged 0-59 living in households where the adults (aged 18-59) work 20% or less of their total work potential during the past year. |
| Income quintile ratio | The ratio of total income received by the 20 % of the population with the highest income (top quintile) to that received by the 20 % of the population with the lowest income (lowest quintile). | Income must be understood as equivalised disposable income. (*) |
| GINI coefficient | Defined as the relationship of cumulative shares of the population arranged according to the level of equivalised disposable income (*), to the cumulative share of the equivalised total disposable income received by them. |  |
| Gross domestic product (GDP) | It is a basic measure of the overall size of a country's economy. Equal to the sum of the gross value added of all resident institutional units engaged in production, plus any taxes on products and minus any subsidies on products. Gross value added is the difference between output and intermediate consumption. | GDP is also equal to: The sum of the final uses of goods and services (all uses except intermediate consumption) measured in purchasers' prices, minus the value of imports of goods and services; the sum of primary incomes distributed by resident producer units. |
| Employment rate | The percentage of [employed persons](https://ec.europa.eu/eurostat/statistics-explained/index.php?title=Glossary:Employed_person) in relation to the comparable total population. For the overall employment rate, the comparison is made with the population of working-age; but employment rates can also be calculated for a particular age group and/or gender in a specific geographical area (for example the males of age 15-24 employed versus total in one [EU](https://ec.europa.eu/eurostat/statistics-explained/index.php?title=Glossary:European_Union_(EU))  Member State). | Covers employees and self-employed persons working in resident production units, i.e. the domestic employment concept.  Employment in persons counts all persons engaged in productive activities. Employment in hours worked refers to all hours actually worked, whether paid or not. |
| Unemployment rate and long-term unemploy-ment rate | The **unemployment rate** is the number of people unemployed as a percentage of the labour force.  **Long term unemployment rate:** Total long-term unemployed population (≥12 months' unemployment; ILO definition) as a proportion of total active population aged 15 years or more. | An unemployed person is defined by Eurostat, according to the guidelines of the International Labour Organization, as:  • Someone aged 15 to 74 (in Italy, Spain, the United Kingdom, Iceland, Norway: 16 to 74 years);  • Without work during the reference week;  • Available to start work within the next two weeks (or has already found a job to start within the next three months);  • Actively having sought employment at some time during the last four weeks. |
| Material deprivation rate | Covers indicators relating to economic strain, durables, housing and environment of the dwelling. | Severely materially deprived persons have living conditions severely constrained by a lack of resources, they experience at least 4 out of 9 following deprivations items: cannot afford i) to pay rent or utility bills, ii) keep home adequately warm, iii) face unexpected expenses, iv) eat meat, fish or a protein equivalent every second day, v) a week holiday away from home, vi) a car, vii) a washing machine, viii) a colour television, or ix) a telephone. |
| Migration and migrant population statistics | **Migration** refers to the number of migrants, people changing their residence to or from a given area (usually a country) during a given time period (usually one year). |  |
| ^a^ The **equivalised disposable income** is the total income of a household, after tax and other deductions, that is available for spending or saving, divided by the number of household members converted into equalised adults; household members are equalised or made equivalent by weighting each according to their age, using the so-called modified [OECD](https://ec.europa.eu/eurostat/statistics-explained/index.php?title=Glossary:OECD) equivalence scale (5).  ***Abbreviations:*** EU, European Union; ILO, International Labour Organization, OECD, Organisation for Economic Co-operation and Development | | |

**Supplementary material references**

1. European Commission. Eurostat -Item 4.2 Standardisation of social variables -Progress report- Meeting of European Directors of Social Statistics: Luxembourg. 2017. <https://circabc.europa.eu/sd/a/7039be8c-a45a-493f-bc49-987e0ba8f798/DSS-2017-Mar-4.2%20Standardisation%20of%20social%20variables%20%20progress%20report.pdf>. Accessed 10 Oct 2019.

2. European Union. Eurostat. European Health Interview Survey (EHIS wave 3). Methodological manual. 2018. <https://ec.europa.eu/eurostat/documents/3859598/8762193/KS-02-18-240-EN-N.pdf/5fa53ed4-4367-41c4-b3f5-260ced9ff2f6>. Accessed 10 Oct 2019.

3. European Union. Social Protection Committee Indicators Sub-group. Portfolio of EU Social Indicators for the Monitoring of Progress Towards the EU Objectives for Social Protection and Social Inclusion. 2015. <https://ec.europa.eu/social/main.jsp?catId=738&langId=en&pubId=7855&furtherPubs=yes>. Accessed 10 Oct 2019.

4. European Commission. Eurostat. <https://ec.europa.eu/eurostat/>. Accessed 15 Nov 2019.

5. Eurostat. Glossary:Equivalised disposable income 2018. <https://ec.europa.eu/eurostat/statistics-explained/index.php/Glossary:Equivalised_disposable_income>. Accessed 29 Jul 2020.
